# Melatonin-mediated FKBP4 downregulation protects against stress-induced neuronal mitochondria dysfunctions by blocking nuclear translocation of GR

**DOI:** 10.1038/s41419-023-05676-5

**Published:** 2023-02-21

**Authors:** Min Jeong Kim, Gee Euhn Choi, Chang Woo Chae, Jae Ryong Lim, Young Hyun Jung, Jee Hyeon Yoon, Ji Yong Park, Ho Jae Han

**Affiliations:** 1 Department of Veterinary Physiology, College of Veterinary Medicine, Research Institute for Veterinary Science, and BK21 Four Future Veterinary Medicine Leading Education & Research Center, Seoul National University, Seoul, 08826 South Korea; 2 Laboratory of Veterinary Biochemistry, College of Veterinary Medicine and Veterinary Medical Research Institute, Jeju National University, Jeju, 63243 South Korea

**Keywords:** Molecular neuroscience, Neurophysiology

## Abstract

The physiological crosstalk between glucocorticoid and melatonin maintains neuronal homeostasis in regulating circadian rhythms. However, the stress-inducing level of glucocorticoid triggers mitochondrial dysfunction including defective mitophagy by increasing the activity of glucocorticoid receptors (GRs), leading to neuronal cell death. Melatonin then suppresses glucocorticoid-induced stress-responsive neurodegeneration; however, the regulatory mechanism of melatonin, i.e., associated proteins involved in GR activity, has not been elucidated. Therefore, we investigated how melatonin regulates chaperone proteins related to GR trafficking into the nucleus to suppress glucocorticoid action. In this study, the effects of glucocorticoid on suppressing NIX-mediated mitophagy, followed by mitochondrial dysfunction, neuronal cell apoptosis, and cognitive deficits were reversed by melatonin treatment by inhibiting the nuclear translocation of GRs in both SH-SY5Y cells and mouse hippocampal tissue. Moreover, melatonin selectively suppressed the expression of FKBP prolyl isomerase 4 (FKBP4), which is a co-chaperone protein that works with dynein, to reduce the nuclear translocation of GRs among the chaperone proteins and nuclear trafficking proteins. In both cells and hippocampal tissue, melatonin upregulated melatonin receptor 1 (MT1) bound to Gαq, which triggered the phosphorylation of ERK1. The activated ERK then enhanced DNA methyltransferase 1 (DNMT1)-mediated hypermethylation of *FKBP52* promoter, reducing GR-mediated mitochondrial dysfunction and cell apoptosis, the effects of which were reversed by knocking down *DNMT1*. Taken together, melatonin has a protective effect against glucocorticoid-induced defective mitophagy and neurodegeneration by enhancing DNMT1-mediated FKBP4 downregulation that reduced the nuclear translocation of GRs.

## Introduction

Stress-inducing levels of glucocorticoid can cause mitochondrial dysfunction, a major early pathological feature of numerous neurodegenerative diseases, by impairing the mitochondrial quality control system, such as mitophagy which finally results in neuronal cell apoptosis [1, 2]. Under stress conditions, glucocorticoid-bound GRs move from the cytosol to the nucleus, triggering stress response signaling pathways such as promoting apoptosis or depression-related gene expression acting as a transcription factor or a co-factor [3–6]. According to our previous research, activated GRs by excessive glucocorticoid directly bind to the PGC1α promoter as a transrepressor, reducing its expression and nuclear translocation, which resulted in downregulation of NIX. NIX, otherwise known as BCL-2/adenovirus E1B 19kda protein-interacting protein 3-like (BNIP3L), is located on the outer membrane of mitochondria acting as a mitophagy receptor interacting with LC3/GABARAP protein and induces clearance of damaged or dysfunctional mitochondria forming mitophagosome. Also, NIX is capable of maintaining basal mitophagy that continuously occurs mostly in energy-demanding tissues such as heart and brain and compensating for other regulators of mitophagy [7]. Especially, we previously found that glucocorticoid inhibits NIX-dependent mitophagy but does not trigger the PINK1-parkin pathway which is not able to compensate for the reduced basal mitophagy [8]. For these reasons, finding a strategy to reduce the nuclear translocation of GRs is thereby important to suppress mitochondrial dysfunction induced by glucocorticoid-induced NIX deficiency. Without a ligand, GR in the cytosolic compartment forms a multiprotein complex with chaperones and co-chaperone proteins including heat shock protein90 (HSP90), FKBP51 (FKBP5), FKBP52 (FKBP4), PP5, and CyP90, most of which contribute to the retention of GR in the cytosol [9–11]. However, ligand-bound GRs disassociate from chaperones and move into the nucleus along the microtubule with the help of some co-chaperone proteins, dynein motor proteins, and nuclear pore complex (NPC) including importins [12, 13]. Accordingly, we assumed that finding the molecules that regulate chaperone proteins and then inhibiting GR nuclear translocation would prevent glucocorticoid-induced defects in NIX-mediated mitophagy and neuronal cell survival.

Glucocorticoid and melatonin are two major circadian hormones that regulate sleep, mood, and appetite [14–16]. During stress, the pineal gland, which produces melatonin, is a target site of glucocorticoid-mediated damage because it also expresses a high density of GRs as other regions such as the hippocampus or prefrontal cortex that mainly regulates stress response [17–19]. The stress-inducing levels of glucocorticoid reduce melatonin release from the pineal gland by downregulating NF-κB, suppressing its beneficial functions such as enhancing mitochondrial health as an antioxidant and protecting from neurodegenerative diseases [20–22]. Thus, we expect that restoring melatonin prevents glucocorticoid-induced mitochondrial defects and neurodegenerative diseases. In addition to the antioxidant function of melatonin, it can inhibit glucocorticoid actions by suppressing GR activity or expression [23]. For example, melatonin can regulate GR activity in mice by impairing HSP90 dissociation from GR or regulating the binding affinity of GR to DNA, thereby preventing GR nuclear translocation [24]. Moreover, melatonin is associated with changes in the density and affinity of the cytoplasmic and nuclear conformation of GRs in rats, but whether melatonin increases or decreases these changes is different depending on the tissue [25]. These findings suggest that protective mechanism of melatonin on mitochondria shows high dependence on glucocorticoid-mediated pathways. Therefore, we assume that melatonin can highly affect the proteins that are related to GR activity or expression, thereby blocking glucocorticoid-mediated pathways.

In the present study, we explored the detailed regulatory mechanism of nuclear transport of GR by melatonin using human neuroblastoma SH-SY5Y cells, a widely used in vitro model to investigate neurodegeneration. In addition, we used ICR mice mimicking a stress-induced model as an in vivo model to determine the recovering effects of melatonin against mitochondrial dysfunction and cognitive impairment induced by high doses of corticosterone. Therefore, the study was conducted using both in vitro and in vivo models to investigate the protective mechanisms of melatonin on stress-induced mitochondria dysfunction and cognitive impairment by regulating the nuclear transport of GR.

## Materials and methods

### Reagents and antibodies

Cortisol, corticosterone, BSA, DAPI, α-tubulin antibody (T6074), melatonin (M5250), PD98059 (027K2176), 5-azacytidine (5-aza, A2385) and carbonyl cyanide m-chlorophenyl hydrazone (CCCP, C2759) were purchased from Sigma Chemical Company (St. Louis, MO, USA). Lamin A/C (sc-376248), β-actin (sc-47778), MT2 (sc-28453), dynein (sc-13524), NUP62 (sc-48373), importin β (sc-137016), BNIP3 (sc-56167), c-MYC (sc-40), IgG mouse (sc-2025), p-ERK (sc-7383), ERK (sc-94), JNK (sc-7345), p-JNK (sc-6254), p-p38 (sc-166182), and PINK1 (sc-33796) antibodies were acquired from Santa Cruz (Paso Robles, CA, USA). MT1 (NBP1-71113), FKBP4 (NB110-96874), FKBP5 (NBP1-84676), LC3 (NB100-2220) and NIX (NBP1-88558) were purchased from Novus Biologicals (Littleton, CO, USA). TOMM20 (ab56783), p-GR (ab55189), DNMT1 (ab19905), DNMT3a (ab23565), and SP1 (ab227383) antibodies were obtained from Abcam (Cambridge, MA, USA). Cleaved caspase 3 (9661 S), DNMT3b (67259 S), GR (12041 S), 5-methylcytosine (5-mc, 28692 S), p38 (9212 S), IgG rabbit (2729 S), and p-c-MYC ser62 (13748 S) antibodies were purchased from Cell Signaling Technology (Danvers, MA, USA). HSP90 antibody (PA3-013) was obtained from Thermo Fisher (Rockford, IL, USA).

### Experimental design of the animal study

Male ICR mice aged 7 weeks were used, in compliance and approval with the Institutional Animal Care and Use Committee (IACUC) of Seoul National University (SNU-211027-5). All experiments of this study were conducted according to IACUC guidelines. ICR mice were delivered from Orient-Bio (Seoul, Korea). After measuring the weight of each mouse, to minimize subjective bias, mice were randomly divided into four groups of the same size, including the control group, the corticosterone-treated group, the corticosterone and melatonin-treated group, and the melatonin-treated group. They were housed for 7 days in controlled specific pathogen-free conditions of 22 °C, 70% relative humidity, a 12 h light: dark cycle, and had unlimited access to a normal diet and water. The hippocampus of mice was mainly used for evaluating glucocorticoid and melatonin effects on mitochondrial function, as the hippocampus has the most abundant GRs among the brain which contributes to its glucocorticoid responsiveness and shows significantly decreased levels of melatonin when exposed to excessive glucocorticoid. Corticosterone was dissolved in the solution containing 50% propylene glycol in PBS and melatonin was dissolved in PBS. Vehicle-treated mice were similarly injected with the solution containing propylene glycol and PBS. Following the previous reports, 10 mg/kg of corticosterone injection increases serum corticosterone levels which reach around 350 ng/ml, known as stress-induced levels of corticosterone [26]. Furthermore, 10 mg/kg of melatonin injection is widely used to restore serum melatonin levels [27]. Therefore, the mice of corticosterone-treated group were administered 10 mg/kg of corticosterone and the melatonin-treated group was intraperitoneally administered 10 mg/kg of melatonin for 7 days. The mice of the corticosterone and the melatonin-treated group were firstly administered 10 mg/kg of melatonin and then given 10 mg/kg of corticosterone 30 min later intraperitoneally for 7 days. After behavioral testing, mice were anesthetized by intraperitoneal injection of alfaxalone (40 mg/kg) with 10 mg/kg xylazine and sacrificed for acquiring serum for hormonal analysis and brain tissue samples.

### Y-maze spontaneous alternation test

The Y-maze behavioral test is used to evaluate spatial memory and learning [28]. Mice instinctively prefer to try a new arm of the Y-maze instead of returning to the one they’ve already explored. Before the Y-maze spontaneous alternation test, the mice were accommodated in the testing room for 2 h to reduce the effects of external environmental stimuli or unintentional stress on their behavior. First, mice were positioned in the Y-maze apparatus. For 8 min, mice were allowed to explore the Y-maze, and the movements of the mice were recorded by using a video camera. The number of alternations was divided by the number of total triads. Animals with a higher alternation percentage tend to have better spatial memory.

### Novel object recognition test

The novel object recognition test is a commonly used mouse behavioral test for assessing object working memory [29]. The mice were habituated for 5 min in an open-field box. After 24 h, the mice were allowed to move freely in the same open field with two comparable items for 10 min. After 4 h, one of the two objects was replaced with a new one, then the mouse was given 10 min to explore the open field. The discrimination index is the most used value for cognitive evaluation.

### Immunocytochemistry

The human neuroblastoma SH-SY5Y cells were fixed with 4% paraformaldehyde for 10 min at room temperature and then incubated in 0.1% Triton X for 5 min. To inhibit the nonspecific binding of antibodies, cells were incubated with 5% normal goat serum for 1 h following the modified protocol from previous report [30]. Next, the cells were treated with primary antibody for 24 h at 4 °C. After being washed with PBS, the cells were applied for 2 h at room temperature with Alexa Fluor 488 or 555-conjugated secondary antibody (1:300) in dark for 2 h at room temperature. Images were acquired by super-resolution radial fluctuations (SRRF) imaging system (Andor Technology, Belfast, UK). The fluorescent intensity analysis and co-localization were performed with Fiji software (developed by Wayne Rasband, National Institutes of Health, Bethesda, MD, USA).

### Immunohistochemistry

Mice were deeply anesthetized and transcardially perfused with PBS, and then with 4% paraformaldehyde in 0.1 M phosphate buffer (pH 7.4). The extracted brains were post-fixed for 2 h in 4% paraformaldehyde and then dehydrated in 30% sucrose in PBS for 24 h at 4 °C following the protocol from our previous report [8]. Serial transverse sections (40 μm) were conducted using a cryostat (Leica Biosystems, Nussloch, Germany). The brain tissues were fixed with 4% paraformaldehyde and blocked with 5% normal goat serum containing 0.1% Triton X-100 for 1 h at room temperature. Brain samples were incubated with primary antibody overnight at 4 °C. Then, the secondary antibody was incubated for 2 h at room temperature. All completed samples were visualized using the SRRF imaging system. Fiji software was used to analyze the fluorescent intensity and determine Pearson’s correlation coefficient values.

### Cell culture

The human neuroblastoma cell line SH-SY5Y has been widely used as a neuronal disease model related to neurodegenerative diseases [31]. Due to its many advantages, SH-SY5Y cell line is especially suitable for molecular mechanism identification studies of neurons due to its differentiation potential into neurons, high homogeneity, and reproducibility. Cells were cultured in high-glucose DMEM supplemented with 10% fetal bovine serum and 1% antibiotics. Fetal bovine serum was purchased from Hyclone (Logan, UT, USA) and antibiotics were purchased from Gibco (Grand Island, NY, USA). Cells were grown in 60 mm and 100 mm culture dishes in an incubator (37 °C, CO_2_ 5%, and air 95%). When the cells reached 70% confluency, the culture medium was replaced with a serum-free medium containing 1% antibiotics for 24 h for starvation. The neuroblastoma cell line SH-SY5Y was purchased from Korea Cell Line Bank (Seoul, Korea). Fetal bovine serum was purchased from Hyclone (Logan, UT, USA) and antibiotics were purchased from Gibco (Grand Island, NY, USA).

### Real-time PCR

RNA was extracted from SH-SY5Y using an RNA extraction Kit (TaKaRa, Otsu, Shinga, Japan, 9767). Reverse transcription PCR was performed using 1 μg of extracted RNA using a Maxime RT-PCR premix kit (iNtRON Biotechnology, Sungnam, Korea, 25801). The cDNA was amplified using a Rotor-Gene 6000 real-time system (Corbett Research, Mortlake, Australia) using a variety of mRNA primers (Table S1) and TB Green Premix EX Taq^TM^ (TaKaRa, RR420A). The real-time PCR was conducted as follows: 10 min at 95 °C, 15 s at 58 °C, and 20 s at 72 °C. To quantify mRNA expression, delta delta Ct method was used and the data were normalized to the *ACTB* gene.

### Western blotting

Cells were collected with RIPA lysis buffer (ATTO Corporation, Tokyo, Japan, WSE-7420) containing protease and phosphatase inhibitor (Thermo Fisher, 78440) and incubated on ice. After homogenization, cell lysates were centrifuged at 13,000 rpm for 20 min. The protein concentration was determined by a BCA assay kit (Thermo Fisher, 23227). The same amount of sample was loaded in an 8–15% SDS-polyacrylamide gel for electrophoresis and then transferred to a polyvinylidene fluoride membrane. The membrane was blocked with 5% skim milk (Gibco, 232100) for 40 min, and the blocked membrane was washed with TBST solution three times every 10 min. Next, the membrane was incubated with primary antibody overnight at 4 °C. After incubation, the membrane was washed with TBST solution three times every 10 min and incubated with horseradish peroxidase-conjugated secondary antibody (1:10,000) at room temperature for 2 h. The western blotting bands were detected by using chemiluminescence (BioRad, Hercules, CA, USA). The quantification of protein bands was performed using the Image J program (developed by Wayne Rasband, National Institutes of Health, Bethesda, MD, USA).

### Subcellular fractionation

Subcellular fractionation was conducted using the EzSubcell subcellular fractionation/extraction kit (ATTO Corporation, WSE-7421) to isolate cytosolic and nuclear proteins. Cytosolic and nuclear samples for western blot analysis were prepared according to the manufacturer’s manual. Lamin A/C and α-tubulin were used as a nuclear and cytosolic protein marker, respectively.

### siRNA transfection

Cells were grown until approximately 70% confluency of the plate. Before treating cortisol and melatonin, cells were incubated with a mixture of 25 nM indicated siRNA and transfection reagent TurboFect (Thermo Fisher, R0531) for 24 h without antibiotics. The medium was then changed to serum-free high glucose DMEM and treated with cortisol or melatonin. Non-targeting (NT) siRNA was used as the negative control. Small interfering RNAs (siRNAs) for *FKBP52*, *MT1*, *DNMT1* and NT were purchased from Bioneer (Daejeon, Korea).

### Measurement of intracellular ROS, mitochondrial ROS (mtROS), and mitochondrial membrane potential

CM-H_2_DCFDA, MitoSOX^TM^ (Thermo Fisher, M36008), and tetramethylrhodamine ethyl ester (TMRE, Sigma Chemical Company, 87917) were used to determine intracellular ROS, mtROS, and mitochondrial membrane potential, respectively [32]. Cells were washed once with PBS and incubated with 1 μM CM-H_2_DCFDA for 15 min, 5 μM MitoSOX^TM^ for 15 min at 37 °C, and 100 nM TMRE for 20 min at 37 °C in dark. After being washed with PBS three times, cells were treated with 0.05% trypsin for 3 min and centrifuged at 1500 × *g* for 5 min. Harvested cells were suspended in 400 μL PBS. Fluorescence intensities of CM-H_2_DCFDA, MitoSOX^TM^, and TMRE were detected by using a Cytoflex flow cytometer (CytoFlex; Beckman Coulter, USA).

### Measurement of Annexin V/PI apoptosis detection

Annexin V and PI staining were used to detect apoptosis of cells using an annexin V/PI apoptosis detection kit (BD Bioscience, BD 556547) [32]. After treatment, cells were suspended in a binding buffer. Next, annexin V-FITC and PI were added to the samples and incubated for 20 min at room temperature in dark. Apoptosis of the samples was detected with flow cytometry (Quanta SC) and data analysis was conducted with CytExpert 2.3 (Beckman Coulter). Annexin V-negative and PI-negative cells were considered viable. Annexin V-negative and PI-positive, Annexin V-positive and PI-positive, Annexin V-positive and PI-negative cells were considered as necrotic, late apoptotic, and early apoptotic cells, respectively. Annexin V-positive cells were considered as apoptotic cells.

### In situ proximity ligation assay

To identify the interaction between GR and FKBP4, Duolink in situ red starter kit mouse/rabbit (Sigma Chemical Company, #DUO92101) was used. After cell fixation with 4% paraformaldehyde, proximity ligation assay probe rabbit anti-GR and mouse anti-FKBP4 antibodies were applied. Duolink^TM^ secondary antibodies were applied for 1 h at 37 °C and then ligase was added. After adding ligase, amplification was done to amplify the signal. The antibodies ligated together if they were close enough (<40 nm). Fluorescent images were visualized with an SRRF imaging system.

### Co-immunoprecipitation

Cells were lysed with the pierce^TM^ IP lysis buffer (Thermo Fisher, 87788) containing a protease inhibitor cocktail. Primary antibodies were immobilized with protein G magnetic beads (Sure Beads, BioRad, 161-4021). Immobilized magnetic beads were incubated with cell lysates for overnight at 4 °C. Beads were washed three times with PBST and eluted with 20 mM glycine buffer (pH 2.0) for 5 min. Then, 1 M phosphate buffer and laemmli sample buffer were added to the samples following the protocol from our previous research [33]. Protein analysis was conducted by western blot where anti-mouse or rabbit IgG antibody was used as a negative control.

### Dot blot analysis

Dot blot analysis was used to determine the DNA methylation status of the SH-SY5Y cells. Using a genomic DNA extraction kit, genomic DNA (gDNA) was extracted (Bioneer, K-3032) [34]. The 100 ng of extracted gDNA was denatured for 10 min at 95 °C and then neutralized on ice for 10 min. On the N^+^ nylon membrane (GE Healthcare, Chicago, IL, USA, RPN203B), gDNA was loaded. After drying the membrane, it was incubated at 80 °C for 2 h. Then, we used 5% skim milk to block membrane for 1 h. After blocking, it was incubated overnight at 4 °C with a 5-mc antibody. The membrane was washed three times with TBST and then incubated for 2 h with HRP-conjugated secondary antibody. To detect DNA, a chemiluminescence detection kit was used (Advansta Inc., K-12045-D50). Image J was used to quantify the dot blot intensity.

### Chromatin immunoprecipitation (ChIP)

ChIP assay was conducted using an EpiQuik^TM^ chromatin immunoprecipitation kit (EpiGentek, Farmingdale, NY, USA, P-2002) following the manufacturer’s instructions. Samples including protein-chromatin complexes were incubated with ChIP grade antibody, the RNA polymerase (RNAPol), and the normal IgG. RNAPol and normal IgG were used as a positive control and negative control, respectively. Sample DNA was acquired by supplied column and amplified by PCR using a designed primer (Table S2). One percent of the sample chromatin extract was used as an input.

### Methylation-specific PCR

EZ DNA methylation-lightning kit was used to analyze the methylation of gDNA by bisulfite treatment [34]. EZ DNA Methylation-Lightning Kit (D5030) was purchased from Kyongshin Scientific company (Seoul, Korea). Methylation-specific PCR was performed to evaluate the methylation status of the *FKBP52* gene in SH-SY5Y cells. Methylation-specific PCR was conducted following the manufacturer’s instructions (Zymo Research, Irvine, CA, USA, D5030). Methylation status was assessed as a relative level of methylation compared to the unmethylated form. The primer sequences for the CpG site of the *FKBP52* promoter are shown in Table S3.

### Melatonin ELISA

For the quantification of melatonin in mice plasma samples, the melatonin ELISA kit was used. Mice blood was collected in an EDTA tube and centrifuged at 4000 × *g* for 5 min to isolate plasma. All procedures of the melatonin ELISA kit were conducted according to the supplier’s protocol. Melatonin ELISA kit (MBS765748) was purchased from MyBioSource company (San Diego, CA, USA).

### Statistical analysis

Statistical analysis and graphing were performed using GraphPad Prism version 6.0 (GraphPad Inc., San Diego, CA, USA) statistical software. The sample size ‘n’ represents the number of biological independent replicates and was kept similar between experimental groups and replicates of experiments. One-way ANOVA (with Dunnett’s multiple comparison test) or two-way ANOVA (with Tukey’s multiple comparison test) were used for analyzing the differences among multiple groups. All quantitative data were expressed as mean ± standard error of the mean (S.E.M.). A result with a *p-*value of <0.05 was considered statistically significant. Using samples with a minimum of three can be acceptable if very low *p* values are observed rather than using a large number of samples with interfering results. Therefore, we set *n* = 5 for each experiment in order to gain statistical power.

## Results

### Melatonin-induced improvement in NIX-dependent mitophagy prevents glucocorticoid-induced neuronal cell apoptosis

**Fig. 1:**
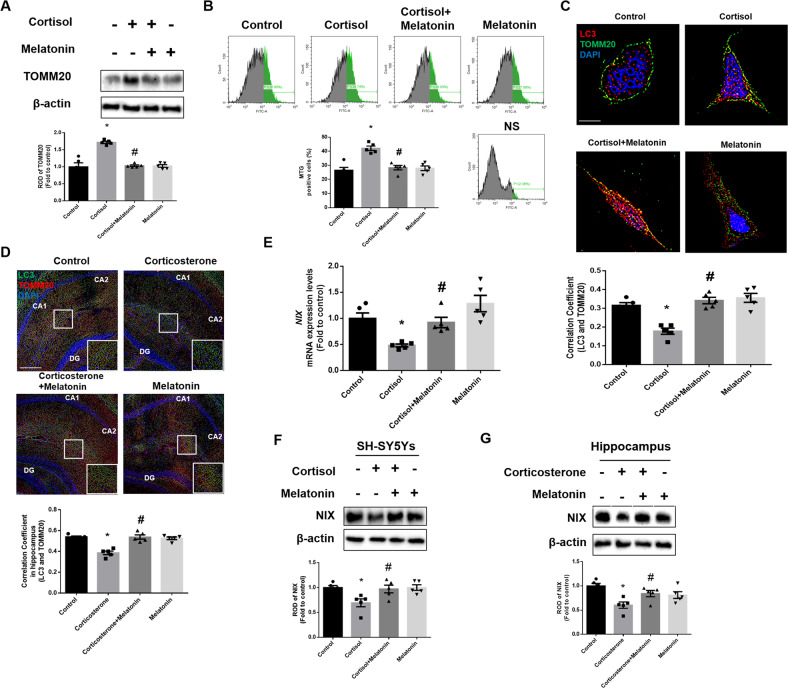
Melatonin enhances glucocorticoid-suppressed mitophagy by recovering NIX. **A**–**C**, **F** SH-SY5Y cells were treated with melatonin (1 μM) for 30 min and then with cortisol (1 μM) for 24 h. **A** Protein levels of TOMM20 were investigated by western blot. Loading control is β-actin. *n* = 5. **B** After incubation, Mitotracker green (200 nM) was stained to detect mitochondrial contents. *n* = 5. **C** SH-SY5Y cells were immunostained with LC3 (red), TOMM20 (green) and DAPI (blue). Scale bars, 10 μm (magnification, ×1000). *n* = 5. **D**, **G** Mice were injected with melatonin (10 mg/kg) and then with corticosterone (10 mg/kg) for 7 days. **D** Slide samples for immunohistochemistry were immunostained with LC3 (green), TOMM20 (red), and DAPI (blue). Scale bars, 140 μm (magnification, ×100). *n* = 5. **E** Cells were treated with melatonin for 30 min and then with cortisol for 12 h. The mRNA expression of *NIX* was analyzed by real time PCR. *n* = 5. **F**, **G** NIX was detected by western blot. Loading control is β-actin. *n* = 5. All blots and immunofluorescence images are representative. The representative images were acquired by SRRF imaging system. All data are presented as a mean ± S.E.M. *p < 0.05 versus control, ^#^*p* < 0.05 versus cortisol or corticosterone. NS means non-staining.

In a previous study, we demonstrated that glucocorticoid suppresses NIX-mediated mitophagy by increasing the nuclear translocation of GR, which then repressed *PPARGC1A* transcription [8]. Then, we investigated whether melatonin reversed the suppressive effects of glucocorticoids on mitophagy and the subsequent neurodegeneration. In humans and rodents, major glucocorticoids, such as cortisol and corticosterone, are released during stress. Therefore, we treated SH-SY5Y cells with cortisol and ICR mice with corticosterone. According to a previous study, 1 μM of cortisol was used in SH-SY5Y cells throughout the study, an amount similar to stress-inducing levels of glucocorticoids in both humans and rodents [35]. To assess the recovering effect of melatonin on mitophagy, we measured mitochondrial contents in SH-SY5Y cells by detecting mitochondria marker TOMM20 in western blot results and cells stained with Mitotracker green. As shown in Fig. 1A, B, melatonin reduced the increased mitochondrial contents by cortisol, representing impaired mitophagy. Then, we detected the extent of mitophagy by immunostaining the cells and hippocampal tissue with LC3 and TOMM20. LC3 bridges mitophagosome components with dysfunctional mitochondria and is converted into LC3II form. We have found that co-localization between LC3 and TOMM20 was decreased by cortisol and corticosterone, but recovered with melatonin treatment (Fig. 1C, D). Next, we confirmed whether melatonin recovered mitophagy by selectively increasing NIX expression. Performing real-time PCR, downregulated *NIX* mRNA expression by cortisol was significantly recovered by melatonin (Fig. 1E). Consistently, glucocorticoid-induced NIX downregulation was significantly reversed by melatonin in both SH-SY5Y cells and hippocampal tissue (Fig. 1F, G).

**Fig. 2:**
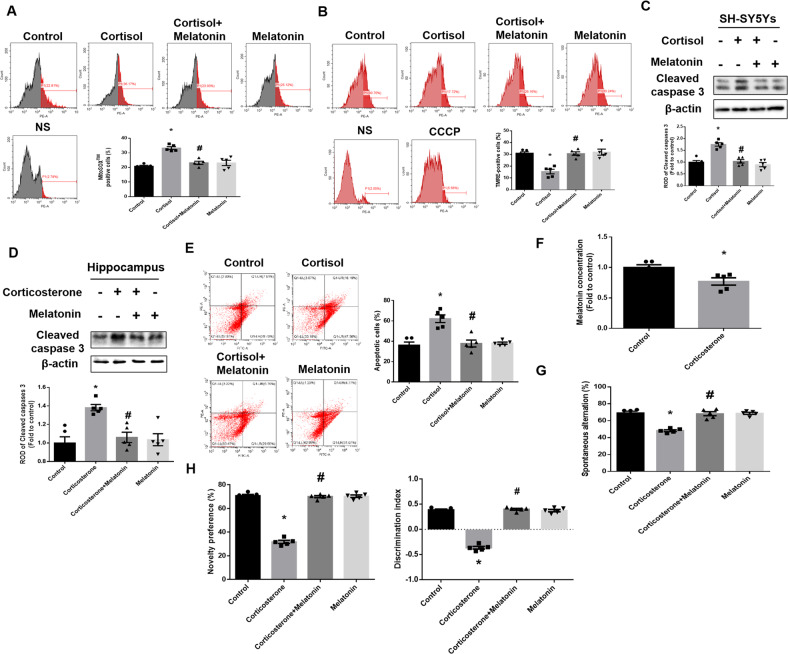
Melatonin protects neuronal cells against glucocorticoid-induced mitochondrial dysfunction and cognitive impairment. **A** SH-SY5Y cells were treated with melatonin (1 μM) for 30 min and then with cortisol (1 μM) for 24 h. The population of MitoSOX™ positive cells was measured by flow cytometry. *n* = 5. **B**, **C** SH-SY5Y cells were treated with melatonin for 30 min and then with cortisol for 48 h. Mitochondrial membrane potential was measured via TMRE staining detected with flow cytometry. *n* = 5. **D**, **F**–**H** Mice were injected with melatonin (10 mg/kg) and then with corticosterone (10 mg/kg) for 7 days. **C**, **D** The expression of cleaved-caspase 3 was detected with western blot. Loading control is β-actin. *n* = 5. **E** SH-SY5Y cells were treated with melatonin for 30 min and treated with cortisol for 72 h. Quantitative analysis of fold changes in apoptotic cells was measured by using annexin V/PI staining with flow cytometry. *n* = 5. **F** Blood was collected from abdominal aorta of mouse groups, and then plasma was isolated. Plasma melatonin level was measured using ELISA. *n* = 5. **G**, **H** To evaluate spatial and object working memory function, mice were subjected to the Y-maze test and novel object recognition test. *n* = 5. All blots and fluorescence images are representative. All data are presented as a mean ± S.E.M. *p < 0.05 versus control, ^#^*p* < 0.05 versus cortisol or corticosterone.

Next, we observed whether melatonin recovered cortisol-induced mitochondrial dysfunction and cell apoptosis resulting from impaired NIX-mediated mitophagy. To assess the effect of melatonin on mtROS accumulation and membrane potential, we stained cells with MitoSOX^TM^ and TMRE, which is a cell permeant mitochondrial superoxide indicator and mitochondrial membrane potential, respectively. Flow cytometry revealed that melatonin reversed cortisol-induced increased mtROS and reduced mitochondrial membrane potential (Fig. 2A, B). Mitochondrial dysfunction resulted in cell apoptosis caused by various pathways such as the activation of intrinsic pathways by cytochrome c release. We then confirmed that melatonin reduced glucocorticoid-induced cleavage of caspase-3 in both SH-SY5Y cells and hippocampal tissue (Fig. 2C, D). Furthermore, the number of annexin V-positive cells, indicating apoptotic cells, was increased following cortisol treatment, whereas melatonin promoted cell survival (Fig. 2E). Then, we determined the recovery effect of melatonin on corticosterone-induced neurodegeneration in mice. As shown in Fig. 2F, melatonin secretion was significantly reduced in mice exposed to excessive corticosterone. Then, we presumed that melatonin restoration can prevent corticosterone-induced neurodegeneration. Neuronal cell death finally triggered behavior changes, especially cognitive deficits when the hippocampus was damaged. Subsequently, the Y-maze alternation test and novel object recognition test were conducted to evaluate spatial and object working memory, respectively. Corticosterone-exposed mice had impaired spatial and object working memory; however, these were restored by melatonin treatment (Fig. 2G, H). Collectively, our results suggested that melatonin recovered glucocorticoid-induced mitochondrial dysfunction, cell death and cognitive impairment by recovering NIX expression.

### Melatonin suppresses glucocorticoid-induced nuclear translocation of GR by downregulating FKBP4

**Fig. 3:**
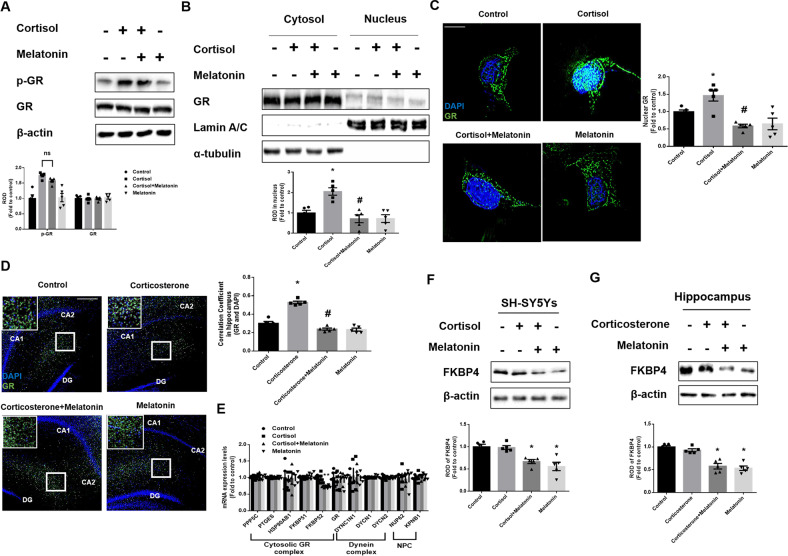
Melatonin downregulates FKBP4, but is not related to phosphorylation of GR. **A**–**C**, **F** SH-SY5Y cells were treated with melatonin (1 μM) for 30 min and then with cortisol (1 μM) for 24 h. **A** The expression of p-GR and GR were detected by western blot. Loading control is β-actin. *n* = 5. **B** The expression of GR protein in subcellular fraction samples was detected by western blotting. Lamin A/C and α-tubulin were used as a nuclear and cytosolic loading control, respectively. *n* = 5. **C** The cells were immunostained with GR (green) and DAPI (blue). Scale bars, 10 μm (magnification, ×1000). *n* = 5. **D**, **G** Mice were injected with melatonin (10 mg/kg) and then with corticosterone (10 mg/kg) for 7 days. **D** Slide samples for immunohistochemistry were immunostained with GR (green) and DAPI (blue). Scale bars, 140 μm (magnification, ×100). *n* = 5. **E** SH-SY5Y cells were treated with melatonin for 30 min and then with cortisol for 12 h. The mRNA expression of regulatory proteins related to cytosolic GR complex, dynein complex, and NPC were analyzed by real time PCR. *n* = 5. **F**, **G** FKBP4 was detected by western blot. Loading control is β-actin. *n* = 5. All blots and immunofluorescence images are representative. The representative images were acquired by SRRF imaging system. All data are presented as a mean ± S.E.M. *p < 0.05 versus control, ^#^*p* < 0.05 versus cortisol or corticosterone.

We assumed that melatonin decreased GR nuclear translocation because glucocorticoid downregulated NIX expression via the GR-PGC1α-NIX axis [8]. GR acts as hormone-dependent transcriptional factors regulating the expression of glucocorticoid-responsive genes when translocated into the nucleus [36]. The phosphorylation of GR on Ser211 increases the level of transcriptional activity because it facilitates nuclear translocation and binding affinity to GR [37–39]. We thereby detected both phosphorylation, the active form of GR and the total GR expression. Our data showed no change in GR expression, but cortisol increased GR phosphorylation. Meanwhile, melatonin did not reduce cortisol-induced phosphorylation of GR, suggesting that melatonin did not participate in post-translational modification of GR (Fig. 3A). Then, we assumed that melatonin reduced the nuclear import of GR in other mechanisms. We found that melatonin reduced the nuclear import of GR in both western blot and immunofluorescence staining results (Fig. 3B, C). In addition, we also confirmed that nuclear GR was increased by corticosterone in immunostaining results of the hippocampus, which was reduced by melatonin (Fig. 3D). We then screened the expression of potential proteins that affect GR nuclear translocation. Key molecules were searched by dividing them into three major categories: chaperone proteins that form a complex with GR in the cytosol, dynein complexes that move along with microtubules to facilitate nuclear trafficking of GR, and the NPC which GR passes to act as a transcription factor. In our data, melatonin only reduced mRNA expression of *FKBP52*, which is known as a co-chaperone protein that enters the nucleus with the help of dynein and facilitates GR trafficking into the nucleus (Fig. 3E). Consistently, FKBP4 expression detected by western bot was only significantly downregulated by melatonin treatment in both SH-SY5Y cells and hippocampus (Fig. 3F, G). Accordingly, we focused on the role of FKBP4 in GR trafficking and how it changes with melatonin.

**Fig. 4:**
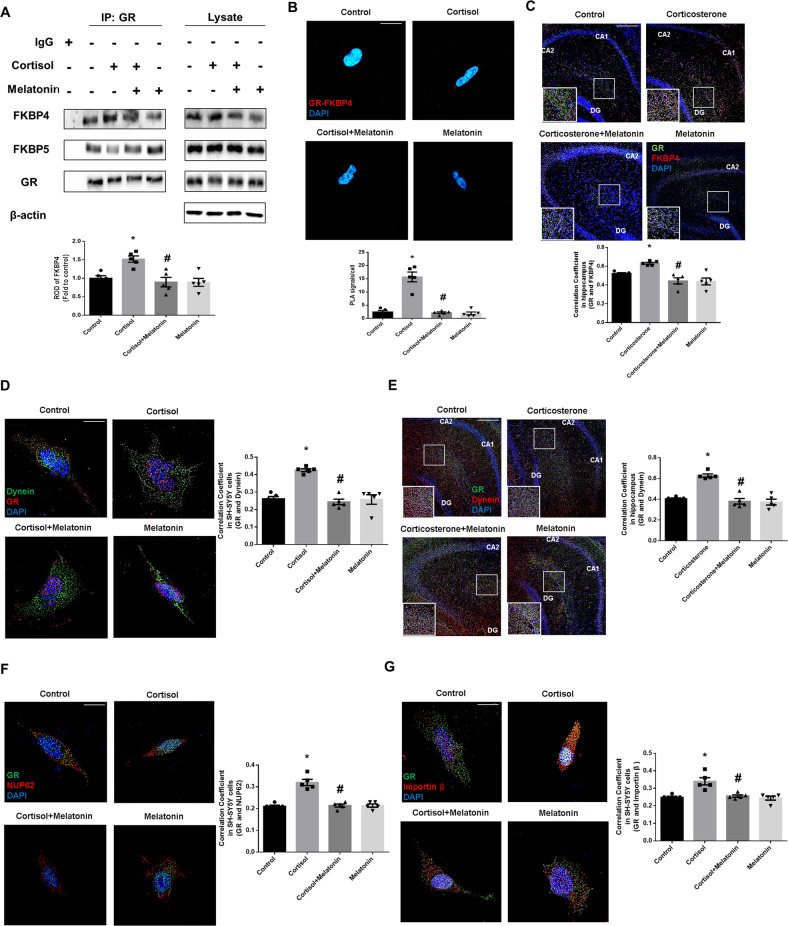
Melatonin-mediated FKBP4 downregulation blocks dynein-dependent GR nuclear translocation. **A**, **B**, **D**, **F**, **G** SH-SY5Y cells were treated with melatonin (1 μM) for 30 min and then with cortisol (1 μM) for 24 h. **A** GR was co-immunoprecipitated with FKBP4 and FKBP5. The level of FKBP4 protein in immunoprecipitated samples was quantified. *n* = 5. **B** The interaction between GR and FKBP4 was investigated via proximity ligation assay. Scale bars, 10 μm (magnification, ×1000). *n* = 5. **C**, **E** Mice were injected with melatonin (10 mg/kg) and then with corticosterone (10 mg/kg) for 7 days. **C** Slide samples for immunohistochemistry were immunostained with GR (green), FKBP4 (red) and DAPI (blue). Scale bars, 140 μm (magnification, ×100). *n* = 5. **D** The cells were immunostained with GR (red), dynein (green) and DAPI (blue). Scale bars, 10 μm (magnification, ×1000). *n* = 5. **E** Slide samples for immunohistochemistry were immunostained with dynein (red), GR (green), and DAPI (blue). Scale bars, 140 μm (magnification, ×100). *n* = 5. **F** The cells were immunostained with GR (green), NUP62 (red), and DAPI (blue). Scale bars, 10 μm (magnification, ×1000). *n* = 5. **G** The cells were immunostained with GR (green), importin β (red), and DAPI (blue). Scale bars, 10 μm (magnification, ×1000). *n* = 5. All blots and immunofluorescence images are representative. The representative images were acquired by SRRF imaging system. All data are presented as a mean ± S.E.M. *p < 0.05 versus control, ^#^*p* < 0.05 versus cortisol or corticosterone.

Without ligand, GR binds to FKBP5 to retain itself in the cytosolic compartment. Conversely, when the ligand is bound, the co-chaperone protein FKBP4 allows GR to enter the nucleus attaching to dynein. In other words, GR moves into the nucleus in a ligand-dependent manner when binding to FKBP5 is changed into FKBP4. Thus, we further investigated the binding status of FKBP4 or FKBP5 to GR. Compared with the interaction between FKBP5 and GR, the binding of FKBP4 to GR increased following cortisol treatment, which was reduced by melatonin (Fig. 4A). Performing proximity ligation assay and immunostaining, we also observed that cortisol-induced increased co-localization between GR and FKBP4 was reduced with melatonin treatment (Fig. 4B, C). Collectively, we assumed that melatonin-induced FKBP4 downregulation would prevent dynein-mediated nuclear translocation of GR. To determine whether reduced FKBP4 affects the dynein-dependent nuclear translocation of GR, we checked the co-localization between GR and dynein. Our data showed that GR-dynein interaction was increased with glucocorticoids, which was suppressed by melatonin in both SH-SY5Y cells and hippocampal tissue (Fig. 4D, E). In addition, we also checked the interaction of GR with NUP62 and importin β. Our data showed that the interaction of both NUP62 and importin β with GR was increased by glucocorticoids, which were both inhibited by melatonin in SH-SY5Y cells (Fig. 4F, G). Taken together, due to the inhibitory effect of melatonin on FKBP4 expression, decreased binding between GR and FKBP4 leads to suppressed GR nuclear translocation.

### Melatonin downregulates FKBP4 by promoting hypermethylation through DNMT 1 upregulation

**Fig. 5:**
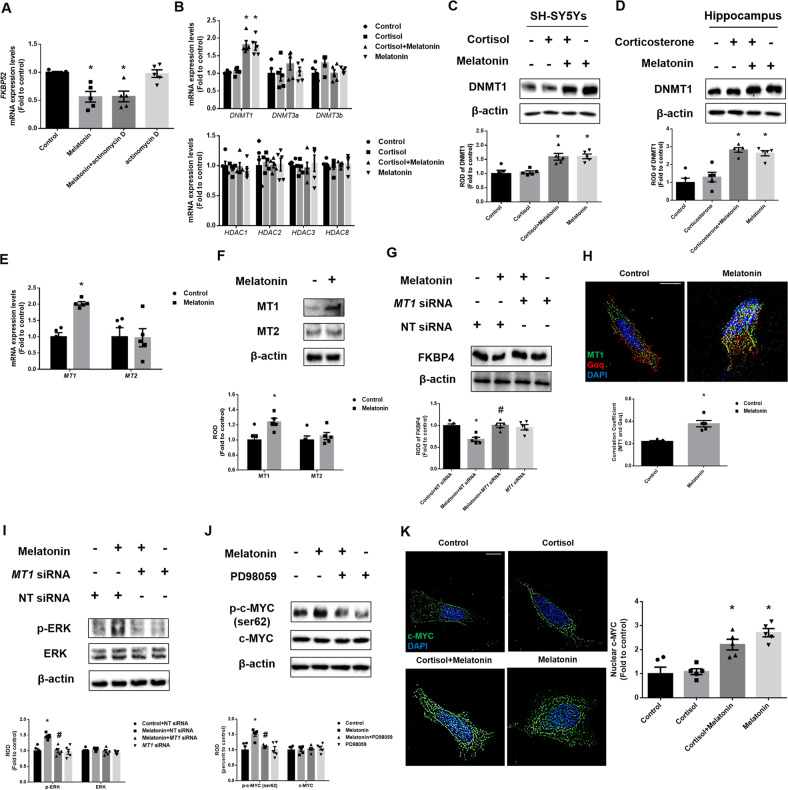
Melatonin upregulates DNMT1 via MT1/ERK/c-MYC axis. **A** The cells were treated with actinomycin D (500 ng/ml) 30 min before treating melatonin for 12 h. The *FKBP52* mRNA expression was analyzed by real-time PCR. *n* = 5. **B**, **K** SH-SY5Y cells were treated with melatonin (1 μM) for 30 min and then with cortisol (1 μM) for 12 h. **B** The mRNA expression of epigenetic-regulated genes was analyzed by real time PCR. *n* = 5. **C** SH-SY5Y cells were treated with melatonin for 30 min and then with cortisol for 24 h. **D** Mice were injected with melatonin (10 mg/kg) and then with corticosterone (10 mg/kg) for 7 days. **C**, **D** DNMT1 expression was detected by western blot. Loading control is β-actin. *n* = 5. **E**, **F**, **H** SH-SY5Y cells were treated with melatonin for 12 h. **E** The *MT1* and *MT2* mRNA expression were analyzed by real time PCR. *n* = 5. **F** MT1 and MT2 levels were detected by western blot. Loading control is β-actin. *n* = 5. **G** The cells were transfected with NT or *MT1* siRNA 24 h before melatonin treatment for 24 h. FKBP4 levels were detected by western blot. Loading control is β-actin. *n* = 5. **H** The cells were immunostained with MT1 (green), Gαq (red) and DAPI (blue). Scale bars, 10 μm (magnification, ×1000). *n* = 5. **I** The cells were transfected with NT or *MT1* siRNA 24 h before melatonin treatment for 12 h. The p-ERK and ERK levels were investigated by western blot. Loading control is β-actin. *n* = 5. **J** PD98059 (50 μM) was treated 30 min before melatonin treatment for 12 h. The p-c-MYC and c-MYC levels were detected by western blot. Loading control is β-actin. *n* = 5. **K** The cells were immunostained with c-MYC (green) and DAPI (blue). Scale bars, 10 μm (magnification, ×1000). *n* = 5. All blots and immunofluorescence images are representative. The representative images were acquired by SRRF imaging system. All data are presented as a mean ± S.E.M. *p < 0.05 versus control or control + NT siRNA, ^#^*p* < 0.05 versus melatonin or melatonin + NT siRNA.

We then investigated by which factors melatonin downregulates FKBP4 expression. First, to determine whether the expression is regulated by transcription factors, we checked the mRNA levels of *FKBP52* by treating actinomycin D, which is known as a transcription factor inhibitor. As shown, melatonin-induced downregulation in *FKBP52* mRNA expression was maintained in a reduced state even with actinomycin D treatment (Fig. 5A). Therefore, we focused on the epigenetics of downregulating FKBP4 and screened for relevant epigenetics-related proteins such as histone deacetylase (HDAC) or DNMT. Isoforms of HDAC class I including HDAC 1, 2, 3, and 8 are highly expressed in brain regions [40]. In general, there are 3 types of DNMTs in the body; DNMT1, DNMT3a, and DNMT3b. Thus, we checked the expressions of HDAC class I proteins and three major DNMTs. Our PCR data revealed that the reduction of FKBP4 expression may be mainly due to DNMT1 upregulation (Fig. 5B). We thereby confirmed the DNMT1 expression both in SH-SY5Y cells and the hippocampus. Western blot results showed that the DNMT1 protein level was increased by melatonin, but there were no changes in its expression when exposed to glucocorticoid treatment (Fig. 5C, D). Then, we determined which type of melatonin receptor mainly upregulates DNMT1. In mammals, there are two major melatonin receptors MT1 and MT2. As shown, melatonin increased the expression of *MT1* mRNA, whereas that of *MT2* was unchanged (Fig. 5E). However, cortisol did not change the expression of MT receptors (Supplemental Fig. S1). Moreover, western blotting results revealed that melatonin increased the expression levels of MT1 proteins (Fig. 5F). Furthermore, we confirmed that *MT1* knockdown reduced the effects of melatonin on FKBP4 downregulation. As shown in Fig. 5G, we confirmed that FKBP4 was downregulated by mediating MT1-dependent pathways of melatonin. MT receptors belong to the G-protein coupled receptor, especially known to bind to Gαq [41, 42]. Moreover, immunofluorescence confirmed that melatonin increased the interaction between Gαq and MT1 (Fig. 5H). The Gαq-mediated pathway activates MAPK by PKC-dependent pathways [43]. The mammalian MAPK includes three subfamilies ERK, JNK, and p38. To determine which MAPKs are affected by melatonin, we performed western blotting. Our results showed with melatonin treatment, ERK phosphorylation was increased, whereas other MAPK activities were not changed (Supplemental Fig. S2). In addition, ERK activation was reduced through *MT1* knockdown (Fig. 5I). Among the transcription factors phosphorylated by ERK, c-MYC is known to regulate DNMT1, which suggests that melatonin increased c-MYC phosphorylation in an ERK-dependent manner [44]. In addition, we observed whether ERK activates serine 62 of c-MYC, a transcription factor that regulates DNMT1 expression. Our data showed that melatonin increased phosphorylated c-MYC at serine 62 (Supplemental Fig. S3). Then, we confirmed that phosphorylated c-MYC was reduced by pretreatment with the ERK inhibitor PD98059 (Fig. 5J). Furthermore, we performed ICC to confirm that melatonin enhanced the nuclear translocation of c-MYC (Fig. 5K). Altogether, DNMT1, a protein that may inhibit FKBP4 expression, was upregulated by melatonin through the MT1/ERK/c-MYC pathway.

**Fig. 6:**
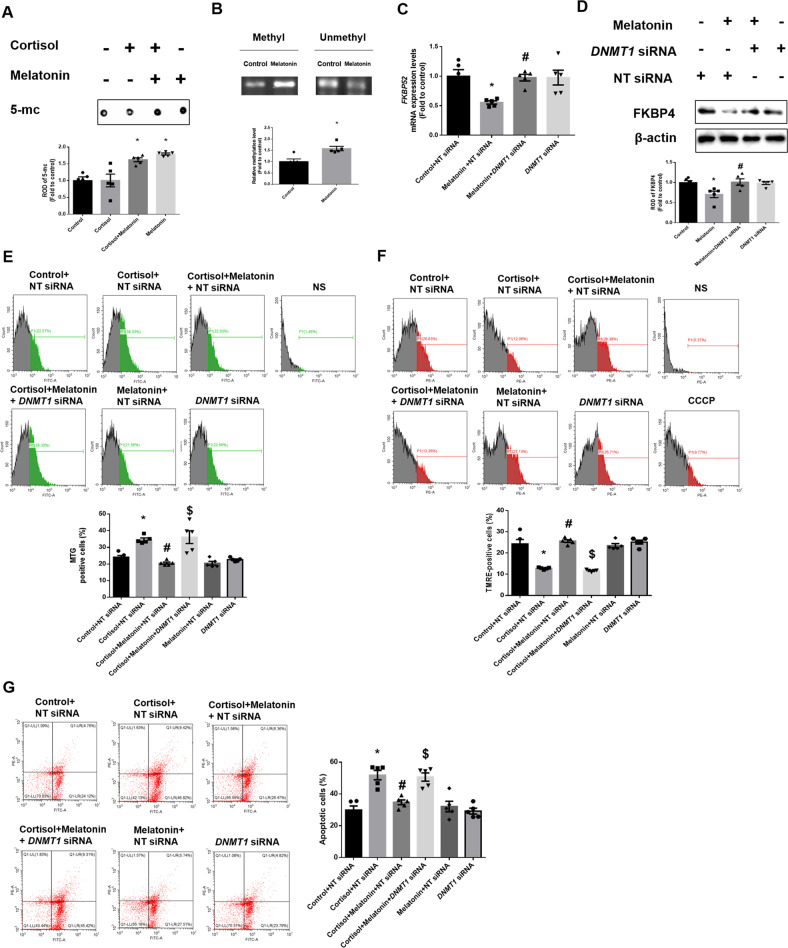
DNMT1-mediated epigenetic downregulation of FKBP4 by melatonin reverses cortisol-induced mitochondrial dysfunction. **A**, **B** SH-SY5Y cells were treated with melatonin (1 μM) for 30 min and then with cortisol (1 μM) for 12 h. **A** Dot blot assay was performed through DNA extraction. The methylation level was confirmed through 5-mc antibody. *n* = 5. **B** The methylation status of the CpG region of the *FKBP52* was confirmed in extracted gDNA. *n* = 5. **C** The cells were transfected with NT siRNA or *DNMT1* siRNA for 24 h before melatonin treatment for 12 h. The mRNA expression of *FKBP52* were analyzed by quantitative real time PCR. *n* = 5. **D** The cells were transfected with NT siRNA or *DNMT1* siRNA for 24 h before melatonin treatment for 24 h. FKBP4 levels were detected by western blot. Loading control is β-actin. *n* = 5. **E** The cells were transfected with NT siRNA or *DNMT1* siRNA for 24 h before melatonin or cortisol treatment for 24 h. Flow cytometry was used to measure mitochondrial mass by Mitotracker green staining. *n* = 5. **F** The cells were transfected with NT siRNA or *DNMT1* siRNA for 24 h before melatonin or cortisol treatment for 48 h. Flow cytometry was used to detect mitochondrial membrane potential by TMRE staining. *n* = 5. **G** The cells were transfected with NT siRNA or *DNMT1* siRNA 24 h before melatonin or cortisol treatment for 72 h. Annexin V/PI staining was performed to detect cell apoptosis. *n* = 5. All blots and fluorescence images are representative. All data are presented as a mean ± S.E.M. *p < 0.05 versus control or control + NT siRNA, ^#^*p* < 0.05 versus melatonin + NT siRNA or cortisol + NT siRNA, and ^$^*p* < 0.05 versus cortisol and melatonin + NT siRNA. NS means non-staining.

Then, we investigated whether melatonin-induced DNMT1 upregulation reduced FKBP4 expression by hypermethylating its promoter. Initially, we checked whether the binding of SP1, known as the transcription factor of *FKBP52*, to its promoter was reduced by melatonin-mediated hypermethylation. In addition, to determine whether FKBP4 downregulation is dependent on DNMT, the DNMT inhibitor 5-aza was used. The results of our ChIP assay in SH-SY5Y cells showed that the binding of SP1 to *FKBP52* promoter was reduced with melatonin treatment, but was reverted by 5-aza (Supplemental Fig. S4). In general, DNMT1 methylates the CpG site of the promoter, which represses gene expression. Therefore, to evaluate the level of methylation in melatonin-treated groups, we conducted a dot blot assay. We conducted this assay using 5-mc, which normally occurs at the CpG site where methylation occurs. In this study, melatonin increased the methylation level, which was not changed in the cortisol-treated group (Fig. 6A). Furthermore, methylation-specific PCR was performed to measure the specific methylation status of the *FKBP4* promoter containing CpG sites. Expectedly, methylation occurred near the promoter of *FKBP52* and was increased by melatonin compared with the control group (Fig. 6B). To confirm that DNMT1 had an inhibitory effect on FKBP4 expression, we transfected cells with *DNMT1* siRNA. FKBP4 transcription and protein expression were decreased by melatonin whereas knockdown of *DNMT1* reversed these effects (Fig. 6C, D). Overall, melatonin-induced DNMT1 inhibits the expression of FKBP4 by methylating its promoter, thereby repressing gene expression. Then, we examined whether melatonin-mediated DNMT1 downregulation improves mitochondrial function by promoting mitophagy. Our Mitotracker green results showed that melatonin reversed cortisol-induced increase in mitochondrial contents, but these effects were abolished by the *DNMT1* knockdown (Fig. 6E). Furthermore, cortisol-induced reduction of mitochondrial potential was reversed by *DNMT1* knockdown (Fig. 6F). SH-SY5Y cells underwent significant apoptosis when exposed to cortisol but were recovered by melatonin, the effect of which was reduced by *DNMT1* knockdown (Fig. 6G). In conclusion, glucocorticoid-induced mitophagy impairment and subsequent mitochondrial homeostasis were recovered by blocking FKBP4-dependent nuclear translocation of GRs by melatonin-induced DNMT1 expression.

## Discussion

Our study provides evidence that melatonin protected both SH-SY5Y cells and hippocampal tissue from glucocorticoid-induced mitochondrial dysfunction and neuronal cell death by blocking GR nuclear translocation through MT1/DNMT1-mediated FKBP4 downregulation. Melatonin and cortisol are well-known circadian rhythm-related hormones that have opposite functions toward sleep [45]. However, when stress activates the hypothalamic-pituitary-adrenal axis leading to high levels of serum cortisol in humans, melatonin release is suppressed and its antioxidant mechanism is impaired, which can then cause neuronal mitochondrial dysfunction and is related to numerous neurodegenerative diseases [46–48]. Consistent with these studies, this study revealed that glucocorticoid-induced mitophagy impairment and mitochondrial dysfunction, followed by neuronal cell apoptosis, were well reversed by melatonin treatment in both SH-SY5Y cells and mouse hippocampal tissue. Melatonin protects against mitochondrial damage of neurons and then rescues the brain from neurodegenerative diseases, such as Alzheimer’s disease, enhancing upregulating activities of major antioxidant enzymes such as superoxide dismutase, inhibiting the mitochondria permeability transition pore, and promoting mitophagy for clearing dysfunctional mitochondria [49–52]. However, suppressing glucocorticoid-induced neurodegenerative pathogenesis by melatonin would be sufficient to restore mitochondrial function and protect neurons from glucocorticoid-mediated neuronal apoptosis. Previous studies have reported that melatonin enhances mitochondrial function by inducing mitophagy, a process of removing damaged mitochondria, and improves cell survival by activating parkin translocation or increasing PINK1 expression [30, 53]. These studies showed that high glucose exposure or cardiomyopathy resulted in the membrane potential of mitochondria was decreased in these pathogenic conditions where melatonin upregulated PINK1 more efficiently whereas glucocorticoid exposure did not trigger membrane potential change in neuronal cells, where melatonin action on PINK1-mediated mitophagy could not effectively restore mitochondrial function. Following our assumption, in our results, melatonin did not solely play an important role in enhancing PINK1-dependent mitophagy or other receptor-mediated mitophagy, suggesting that the action of melatonin can highly depend on abundant glucocorticoid. Instead, melatonin specifically recovered NIX expression reduced by excessive glucocorticoids, indicating a possibility that melatonin repressed the GR-PGC1α-NIX axis, as demonstrated by our previous study. Melatonin may induce mitophagy-associated regulators except NIX. In fact, several studies have revealed that melatonin enhances mitophagy by the PINK1-parkin pathway or autophagy-related genes in brain ischemia, acute brain injury, or Parkinson’s disease models all of which are quite different from glucocorticoid-induced neurodegeneration [52, 54]. We did not screen for these pathways in other brain regions including substantia nigra or cortex, meaning that melatonin can alleviate glucocorticoid-induced impaired mitophagy by increasing PINK1-parkin pathways in these regions. However, we could not detect this phenomenon in hippocampal neurons or SH-SY5Y cells. From these findings, we can conclude that melatonin has a distinct mechanism to recover NIX-mediated pathways in SH-SY5Y cells and the hippocampus exposed to glucocorticoids. Moreover, we can assume that melatonin exerts various actions on mitophagy depending on the brain regions or in the presence of high levels of glucocorticoids. Specifically, since the hippocampus is the brain region that has the most GR, it is most affected by glucocorticoids where melatonin-mediated mitophagy and cell survival protection against glucocorticoid action can be more developed in this region. However, whether melatonin simply blocks glucocorticoid action toward mitophagy or whether it has an independent mechanism downregulating NIX from glucocorticoids need further investigation.

Based on our previous study, blocking the activity of GR is important to restore NIX-dependent mitophagy under stress because ligand-bound GR binds to the PGC1α promoter and downregulates its expression to suppress NIX in neuronal cells exposed to high levels of glucocorticoids [8]. Through our results, we insisted that melatonin-mediated suppression on nuclear transport of GRs can solely improve NIX-mediated mitophagy disrupted by glucocorticoids, thereby enhancing mitochondria function and inhibiting neuronal cell death under stress. Several previous studies have demonstrated the neuroprotective effects of melatonin against glucocorticoid toxicity by blocking GR activity [55]. For example, melatonin directly reduced mRNA expression of corticosteroid receptors in the paraventricular nucleus to counteract glucocorticoid-induced dysregulation of the hypothalamic-pituitary-adrenal axis [56]. However, our results showed no changes in GR expression in neuronal cells. In other studies, melatonin suppressed GR nuclear translocation in thymocytes and peripheral blood mononuclear cells by blocking HSP90 activity [23, 24, 57]. From these previous findings, we can then assume that the suppressive effect of melatonin in GR action is tissue-specific. However, the exact mechanism of melatonin on suppressing GR nuclear translocation after cytoplasmic retention of GR by HSP90 is not well explored. Therefore, we screened which factor changed upon melatonin mainly participates in suppressing GR nuclear translocation. Under stress, excessive levels of glucocorticoids bound to GRs, which then facilitates dissociation from heat shock proteins. Then, GRs are phosphorylated by several kinases such as MAPKs and CDKs induced by glucocorticoid, and its form can easily move into the nucleus more than GRs by increasing its binding affinity into the glucocorticoid-responsive element of the DNA [37, 58]. In this study, as melatonin did not reduce cortisol-induced GR phosphorylation, melatonin is not associated with kinase deactivation toward GR. Instead, melatonin reduced the nuclear transport of GR. With help from association or dissociation with several chaperone proteins, ligand-bound GR is then easily transported into the nucleus. After screening for the related molecules, we found that melatonin blocks the mechanism by which GR-bound chaperone protein is converted from FKBP5 to FKBP4, which makes GR enter the nucleus [10, 59]. Interestingly, our data show that among proteins involved in nuclear translocation, FKBP4 expression was only selectively downregulated by melatonin, which was not changed by glucocorticoid treatment. As GR phosphorylation then contributes to FKBP4-GR binding and subsequent GR nuclear translocation, we can conclude that GR phosphorylated by cortisol could not sufficiently bind to FKBP4 for nuclear translocation because of melatonin-induced FKBP4 downregulation [60, 61]. FKBP4-GR complex formation is moved into the nucleus with the help of dynein and NPC. Dynein anchors the FKBP4-GR complex into the microtubule and facilitates its passage into the nucleus under NPC supervision. A previous study reported that among other nuclear pore glycoproteins (NUPs) present in NPC, only NUP62 and importin β affect GR nuclear translocation in human HEK293T cells [62, 63]. According to our results, melatonin reverted the increased co-localization of dynein/NUPs with GR by glucocorticoid. However, melatonin did not change the mRNA expressions of these genes. Melatonin can have a suppressive effect on this co-localization caused by a reduction in the GR-FKBP4 complex. As the reducing effect of melatonin on nuclear translocation of several transcription factors such as Nrf2 are not related to upregulating/downregulating dynein or NUP expression, we assume that melatonin mainly participates in regulating chaperone proteins of GR to inhibit its nuclear translocation [64, 65]. Thus, we proposed that the FKBP4/5 expression ratio can be one of the key regulatory factors for the nuclear retention of steroid receptors. Taken together, we suggest that the inhibitory effects of melatonin on GR nuclear translocation mainly occur by downregulating the FKBP4 complex but not interfering with GR phosphorylation, thereby reducing the co-localization of GRs with dynein, NUP62, and importin β. Therefore, we focused on the downregulating effects of melatonin on FKBP4 to suppress glucocorticoid-induced neurodegeneration.

When adjusted with transcription inhibitor actinomycin D, melatonin-induced FKBP4 downregulation was not reversed, which means that melatonin downregulated *FKBP4* mRNA expression by regulating epigenetics [66]. A previous study suggested that melatonin triggered epigenetic regulation in human diseases including neurodegenerative diseases, which indeed affected DNA methylation by regulating DNMT expression [67]. In agreement with this study, our results also showed that melatonin changed DNMT expression, but it did not trigger a significant change in HDAC expressions. In addition, cortisol can regulate DNMTs in both non-neuronal and neuronal cells [68, 69]. However, our results showed that cortisol is not associated with the expressions of epigenetic-related genes. This can be due to the exposure stage of glucocorticoid; i.e., glucocorticoid tends to permanently induce epigenetic changes during neurogenesis at the early- or juvenile-stages of mouse models, whereas glucocorticoid-induced changes during adults are usually reversible, regulating transcription with GR, which is similar to our cell and mouse models [70]. Then, we asked why melatonin selectively increased DNMT1 expression. In human dental pulp cells, melatonin significantly increased DNMT1, but it did not regulate the expressions of DNMT3a and DNMT3b [71, 72]. Consistently, our results showed that DNMT1 is a potent mediator of melatonin-induced FKBP4 downregulation in SH-SY5Y cells. Then, we investigated the specific mechanism of how melatonin upregulated DNMT1 expression. In the mammalian brain, melatonin downregulates MT2 receptors during peak secretion, while upregulating MT1 [73]. Our data partially agree with this finding in that melatonin increased the expression of MT1, but not the expression of MT2. *MT1* silencing reversed the downregulating effect of melatonin on FKBP4, suggesting that melatonin increases DNMT1 expression in an MT1 receptor-dependent pathway. Cortisol treatment was not involved in MT expression, indicating that excessive cortisol release in the brain does not enhance melatonin-dependent neuroprotective mechanisms, triggering neurodegeneration. A study reported that dexamethasone modulates melatonin-induced MT2 receptor expression by triggering an immune response in splenic tissue [74]. These findings indicate that glucocorticoid reduce the melatonin release or MTs to block the action of melatonin. Thus, melatonin-induced boosting of MT receptor activity can protect neurons from GR-mediated neurodegeneration. Based on these results, we insisted that DNMT1 is regulated in an MT1-dependent manner; then, we further investigated the accurate pathway of regulating DNMT1. Given the interaction between Gαq and MT1, which then activates PKC and acts as a critical component of the circadian effect of melatonin, we screened the downstream signals of PKC [75, 76]. Based on a previous report that PKC activates MAPKs, we investigated which MAPK was activated in our cells [77]. A previous study reported that melatonin activates the MT1/ERK pathway for Schwann cell dedifferentiation and proliferation and enhances osteoblastic differentiation of MC3T3-E1 cells by activating the ERK pathway [78, 79]. Consistent with the results of these previous studies, our data showed that MT1-induced PKC phosphorylates ERK among MAPKs and p-ERK activates c-MYC phosphorylating ser62, which plays a crucial role in upregulating DNMT1 expression [44]. We can then conclude that DNMT1 upregulation by melatonin-induced MT1 activation is an important target to reduce glucocorticoid-induced GR trafficking into the nucleus to downregulate FKBP4. Despite melatonin supplementation, mitochondrial dysfunction and neuronal cell apoptosis were not recovered when DNMT1 was downregulated, suggesting that mitochondria function is regulated by DNMT1. Therefore, we insist that *DNMT1* knockdown does not reduce FKBP4 expression, leading to mitochondria dysfunction. Further study is necessary; if we perform similar experiments with ERK or c-MYC inhibitor, our study will gain further reliability that these molecules play important roles in suppressing GR nuclear translocation. Taken together, FKBP4 downregulation through DNMT1-mediated hypermethylation by melatonin prevents GR from translocating into the nucleus.

**Fig. 7:**
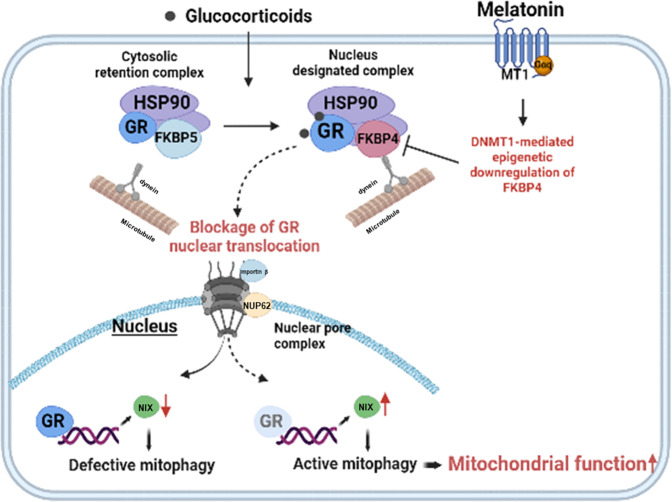
The schematic image is presented. Glucocorticoid induces nuclear translocation of GR and then represses NIX transcription. Melatonin downregulates FKBP4 expression by increasing DNMT1 levels to suppress nuclear translocation of GR, which then restores NIX-mediated mitophagy and neuronal cell survival.

In conclusion, we found the important therapeutic target to treat NIX-dependent mitophagy impairment and subsequent glucocorticoid-induced neuronal cell apoptosis by treating melatonin to downregulate DNMT1-mediated FKBP4 expression (Fig. 7). This study also suggests the therapeutic targeting of FKBPs for the treatment of various stress-mediated neurodegenerative diseases.

## Supplementary information

Supplementary data, legends, and data

Reproducibility checklist

Supplemental Material uncropped WB gel

## Data Availability

The datasets used and/or analyzed during the current study are available from the corresponding author on reasonable request.
