# Supplementary material for: Melatonin-mediated FKBP4 downregulation protects against stress-induced neuronal mitochondria dysfunctions by blocking nuclear translocation of GR
Source: Cell Death Dis. 2023 Feb 21;14(2):146. doi: 10.1038/s41419-023-05676-5 (PMC9943853; doi:10.1038/s41419-023-05676-5)
Supplement: Supplementary file 1 — Supplementary data, legends, and data [file 41419_2023_5676_MOESM1_ESM.docx]

**Supplementary data**

**
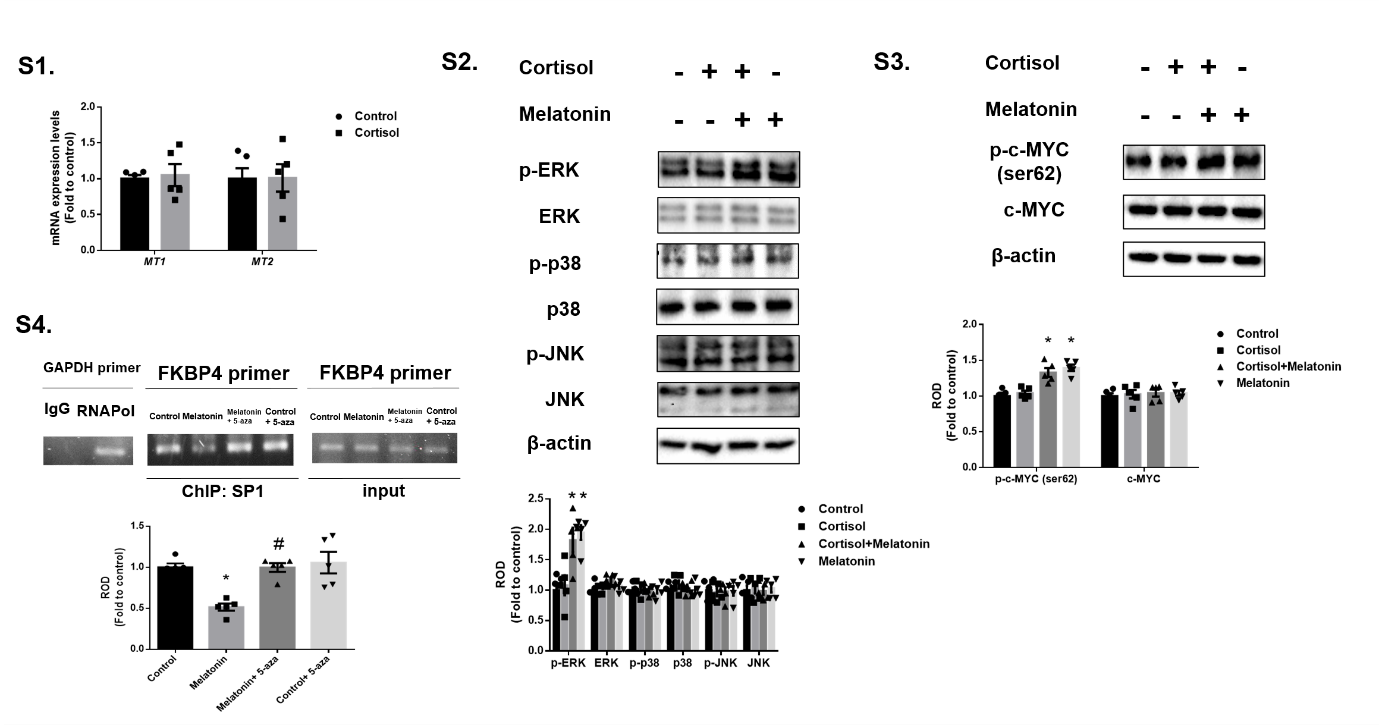
**

**s1.** SH-SY5Y cells were treated with cortisol (1 μM) for 12 h. The mRNA expression of *MT1* and *MT2* were analyzed by real time PCR. *n* = 5. **s2-s3.** SH-SY5Y cells were treated with melatonin (1 μM) for 30 min and then with cortisol for 12 h. **s2.** The expressions of p-ERK, ERK, p-p38, p38, p-JNK, and JNK were detected by western blot. Loading control is β-actin. *n* = 5. **s3.** The expressions of p-c-MYC and c-MYC were detected by western blot. Loading control is β-actin. *n* = 5. **s4.** SH-SY5Y cells were treated with 5-aza (10 μM) for 30 min and then treated with melatonin for 12 h. DNA was immunoprecipitated with IgG, RNAPol and SP1 antibody. The immunoprecipitation and input samples were amplified with primers of *GAPDH* and *FKBP52* gene. *n* = 5. All blots are representative. All data are presented as a mean ± S.E.M. **p* < 0.05 versus control. ^#^*p* < 0.05 versus cortisol or melatonin.

**Table S1 PCR primer**

Primers were created according to the sequence below

| **Target gene** | **Sequence (forward)** | **Sequence (reverse)** |
| --- | --- | --- |
| *NIX* | GGA CTC GGC TTG TTG TGT TG | TAG CTC CAC CCA GGA ACT GT |
| *PPP5C* | CCC AAC TAC TGC GAC CAG AT | CCC GTC ACC TCA CAT CAT TC |
| *PTGES* | CTT CCT TTT CCT GGG CTT CG | GAA GAC CAG GAA GTG CAT CCA |
| *HSP90* | GTA AGC GAT GAT GAG GCT GA | GTC CAG ATG GGC TTT GTT TT |
| *FKBP51* | CTG GAA GTA AAC CCC CAG AA | TGC TTT ATT GGC CTC TTC CT |
| *FKBP52* | GAA TAC AGA CTC GCG GTG AA | CCA TAA GGC AGA TCC AGG TT |
| *GR* | TGG TGT CAC TGT TGG AGG TT | AAA CCT GGT ATT GCC TTT GC |
| *DYNC1N1* | GCC ACC GTC AGT TTT GAC AC | AAA TTG CCT CCA CCA AAC GC |
| *DYCN1* | CGG AAC CTG AAT CTG GAA GAG | TGC AGC TCA TCG TTC ATC TC |
| *DYCN2* | GTG AAG GAG TCA GCC ACA GA | GCT GTT CTT TGT TGC TTC CA |
| *NUP62* | ACA TCG ATG CAC AGC TCA AG | ACT GCA GTG AGT CCA TGT GC |
| *KPNB* | ACC TGC CCA CTT TCC TTG TG | CTC TGC TGA TAT TGT GCC TTG A |
| *DNMT1* | TGA GGC CTT CAC GTT CAA CA | TCC AGG TTG CTG CCT TTG AT |
| *DNMT3a* | TGC CGG AAC ATT GAG GAC AT | TGG CAC ATT CCT CCA ACG AA |
| *DNMT3b* | ACG TCG CTT CTG AAG TGT GT | TCC GCC AAT CAC CAA GTC AA |
| *HDAC1* | CGA TCT GCT CCT CTG ACA AA | CCT TGG TTT TCT CCT CTT CG |
| *HDAC2* | ACT GCC GAA GAA ATG ACA AA | CAC CAC TGT TGT CCT TGG AT |
| *HDAC3* | GGC TTC TGC TAT GTC AAC GA | TCC GTA TTT GTG GAA GGA CA |
| *HDAC8* | GCG TGT TTA TGC AAG CAG TT | TTC CCC TAG GTC CAG TTG AG |

**Table S2 ChIP primer**

Primers were created according to the sequence below

| **Target gene** | **Sequence (forward)** | **Sequence (reverse)** |
| --- | --- | --- |
| SP1 | CAG AAG GAG GCA AGA ACC AA | GTA GGT GGG TCA GGA GCG |

**Table S3 Methylation specific PCR primer**

Primers were created according to the sequence below

| **Target gene** | **Sequence (forward)** | **Sequence (reverse)** |
| --- | --- | --- |
| *FKBP52*-methyl | TCG TTA TTG TTA TTT CGT AGT TTG C | CCT TCT CCT AAA ACC CTC GAA |
| *FKBP52*-unmethyl | TGT TAT TGT TAT TTT GTA GTT TGT GT | CCC TTC TCC TAA AAC CCT CAA |
