# Supplementary material for: Melatonin-mediated FKBP4 downregulation protects against stress-induced neuronal mitochondria dysfunctions by blocking nuclear translocation of GR
Source: Cell Death Dis. 2023 Feb 21;14(2):146. doi: 10.1038/s41419-023-05676-5 (PMC9943853; doi:10.1038/s41419-023-05676-5)

Supplemental Material

Original WB

Fig.1A

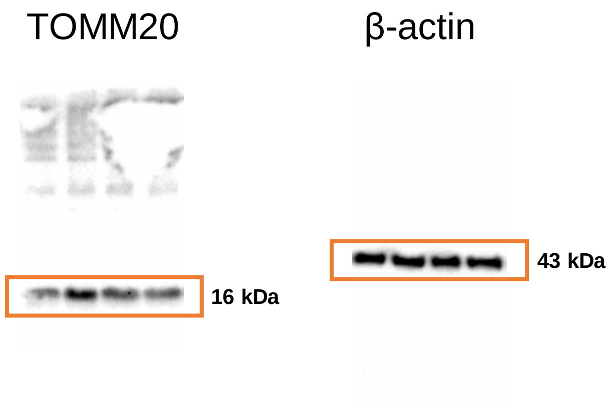

Fig.1F

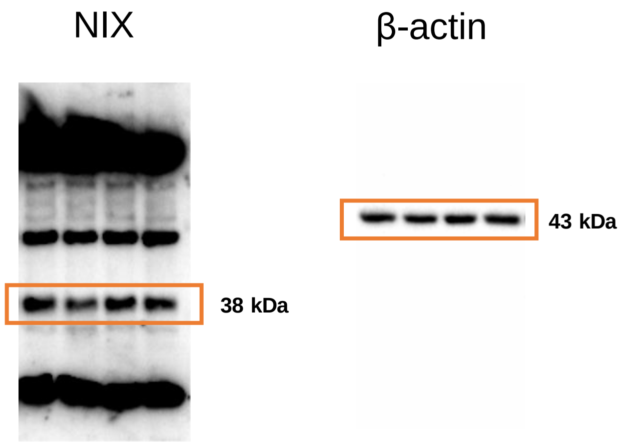

Fig.1G

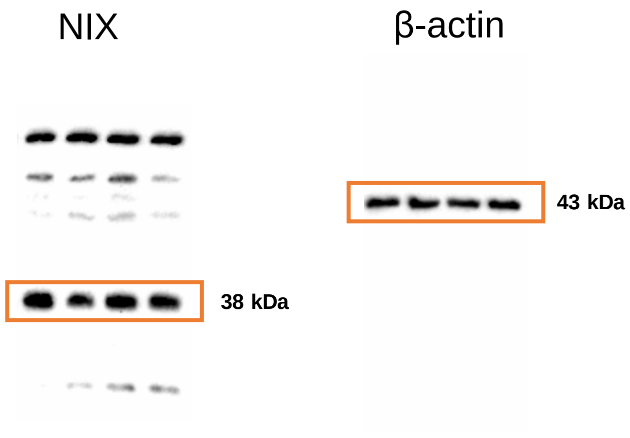

Fig.2C

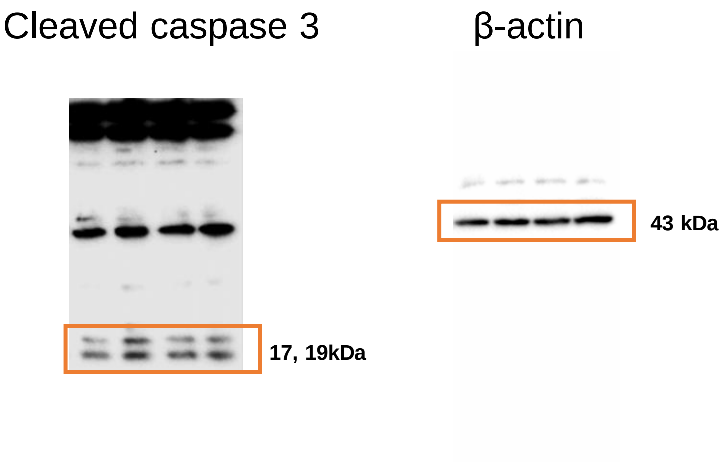

Fig.2D

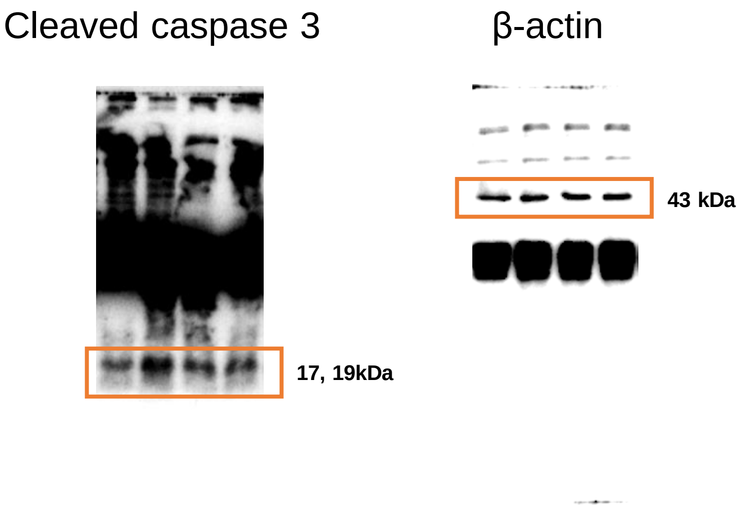

Fig.3A

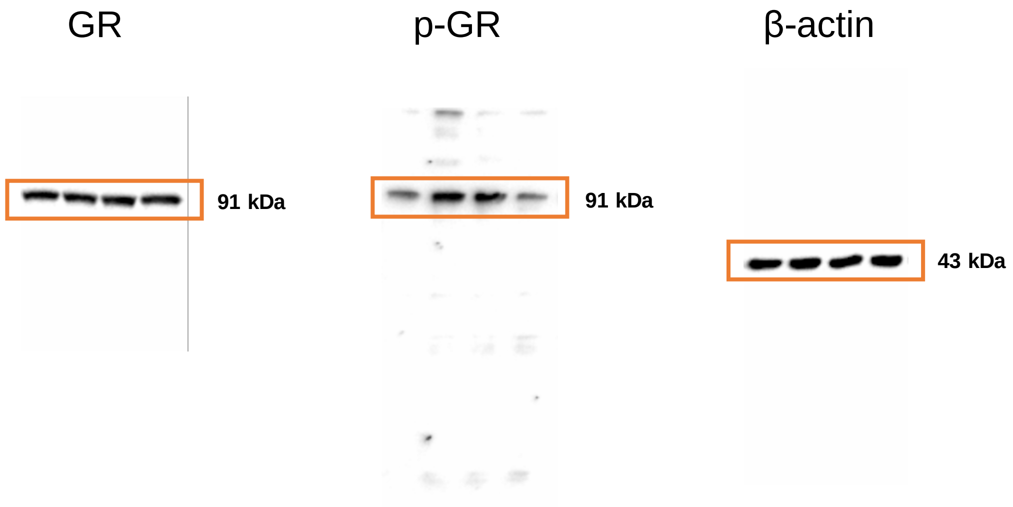

Fig.3B

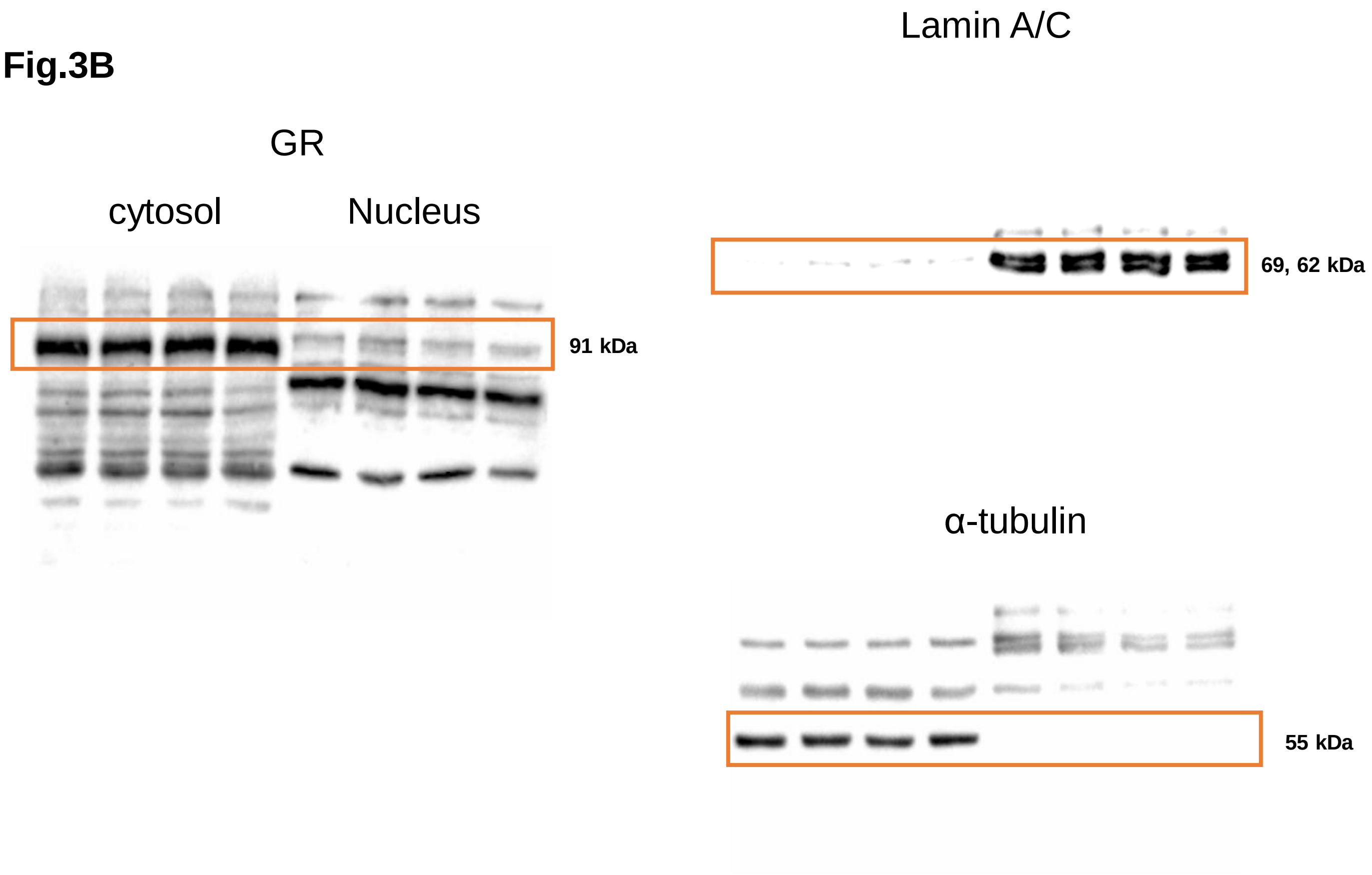

Fig.3F

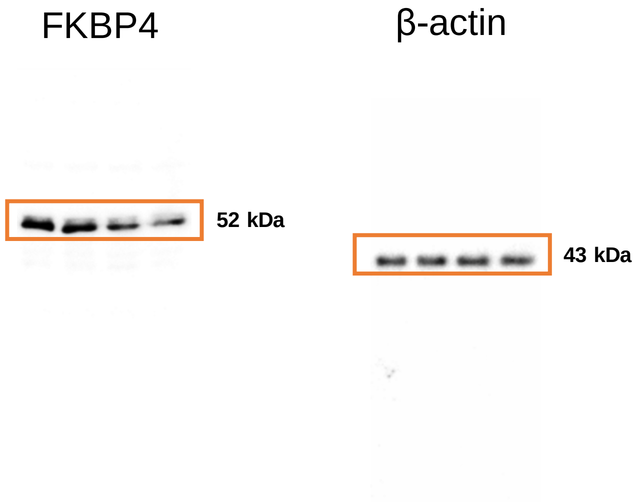

Fig.3G

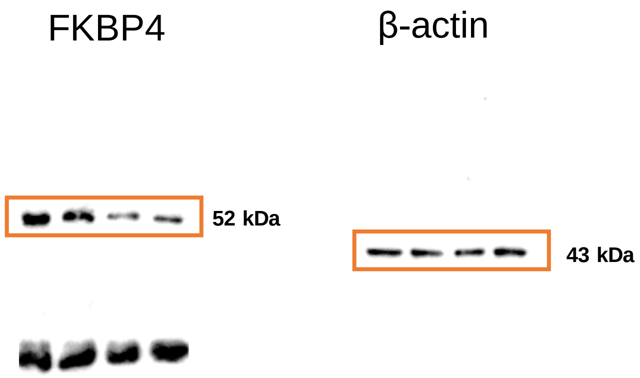

Fig.4A

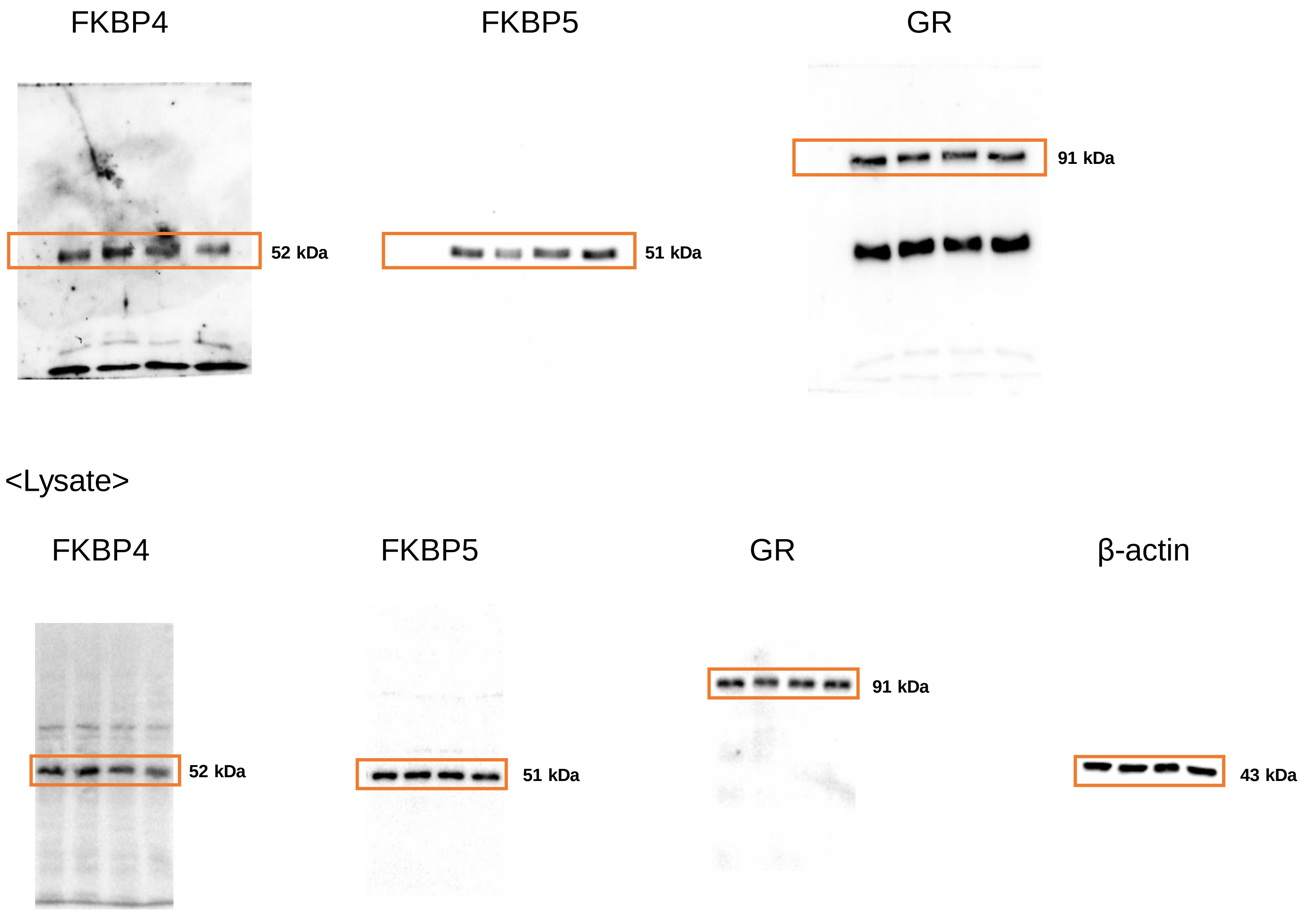
FKBP4
FKBP5
GR
52 kDa
51 kDa
91 kDa
<Lysate>
FKBP4
FKBP5
GR
β-actin
52 kDa
51 kDa
91 kDa
43 kDa

Fig.5C

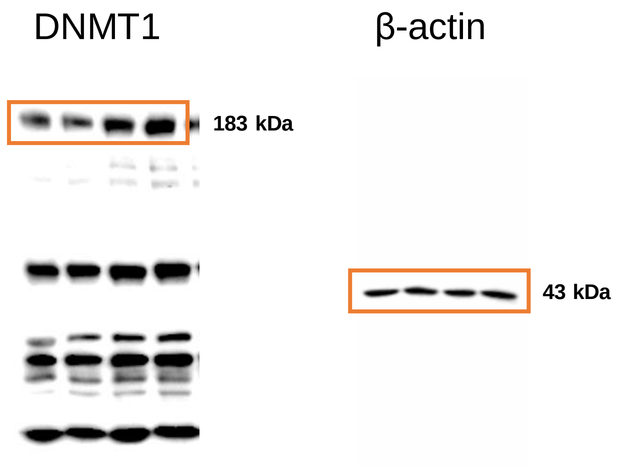

Fig.5D

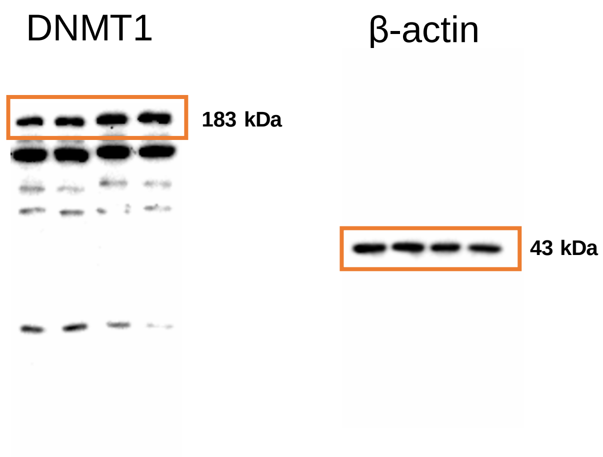

Fig.5F

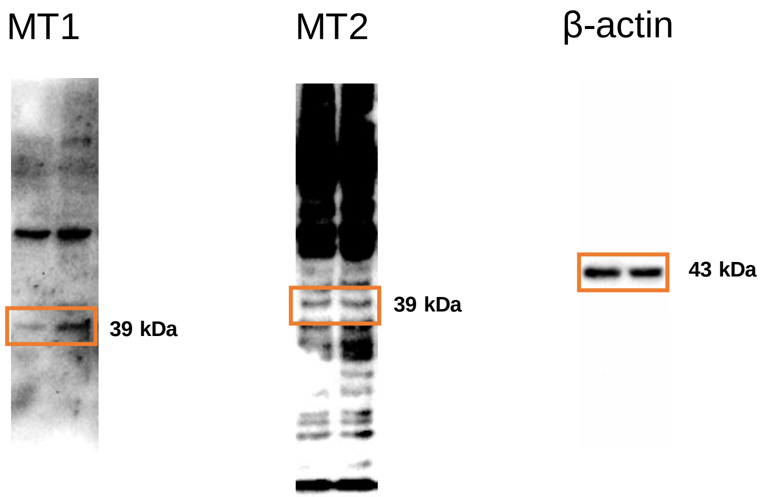

Fig.5G

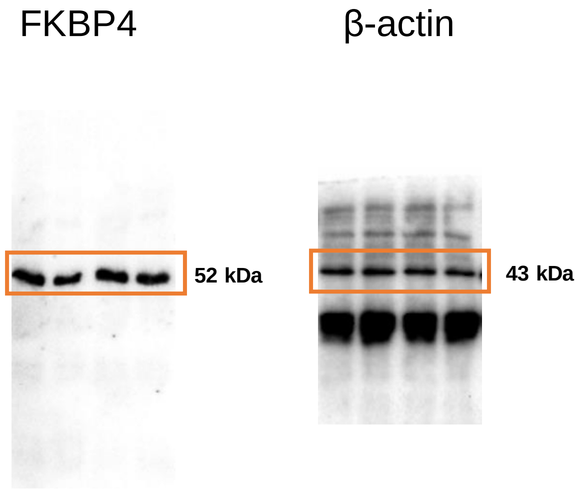

Fig.5I

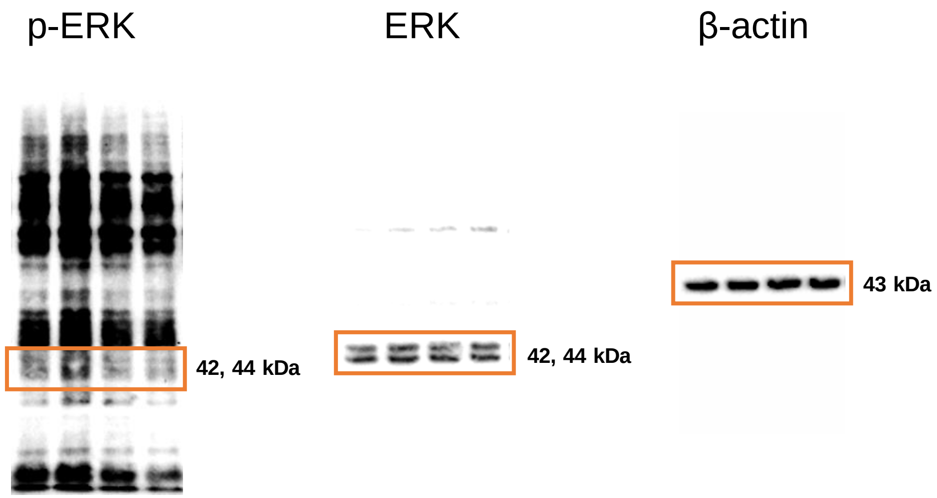

Fig.5J

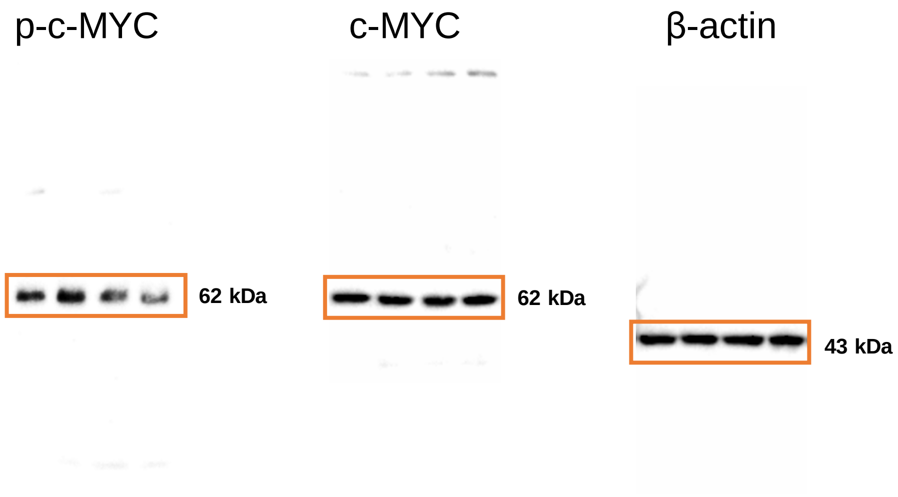

Fig.6D

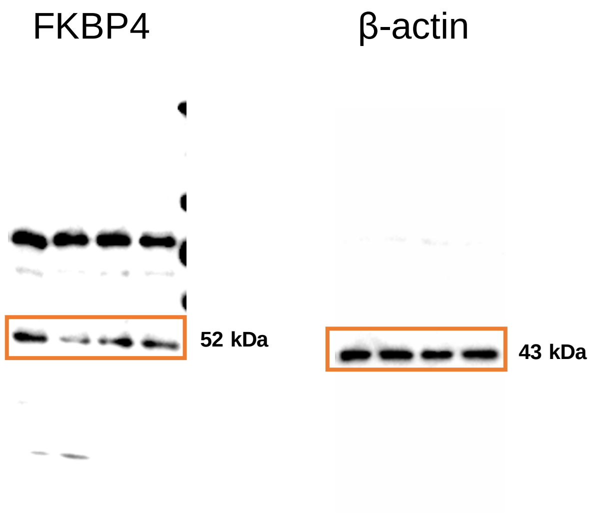

Supplemental Fig S2.

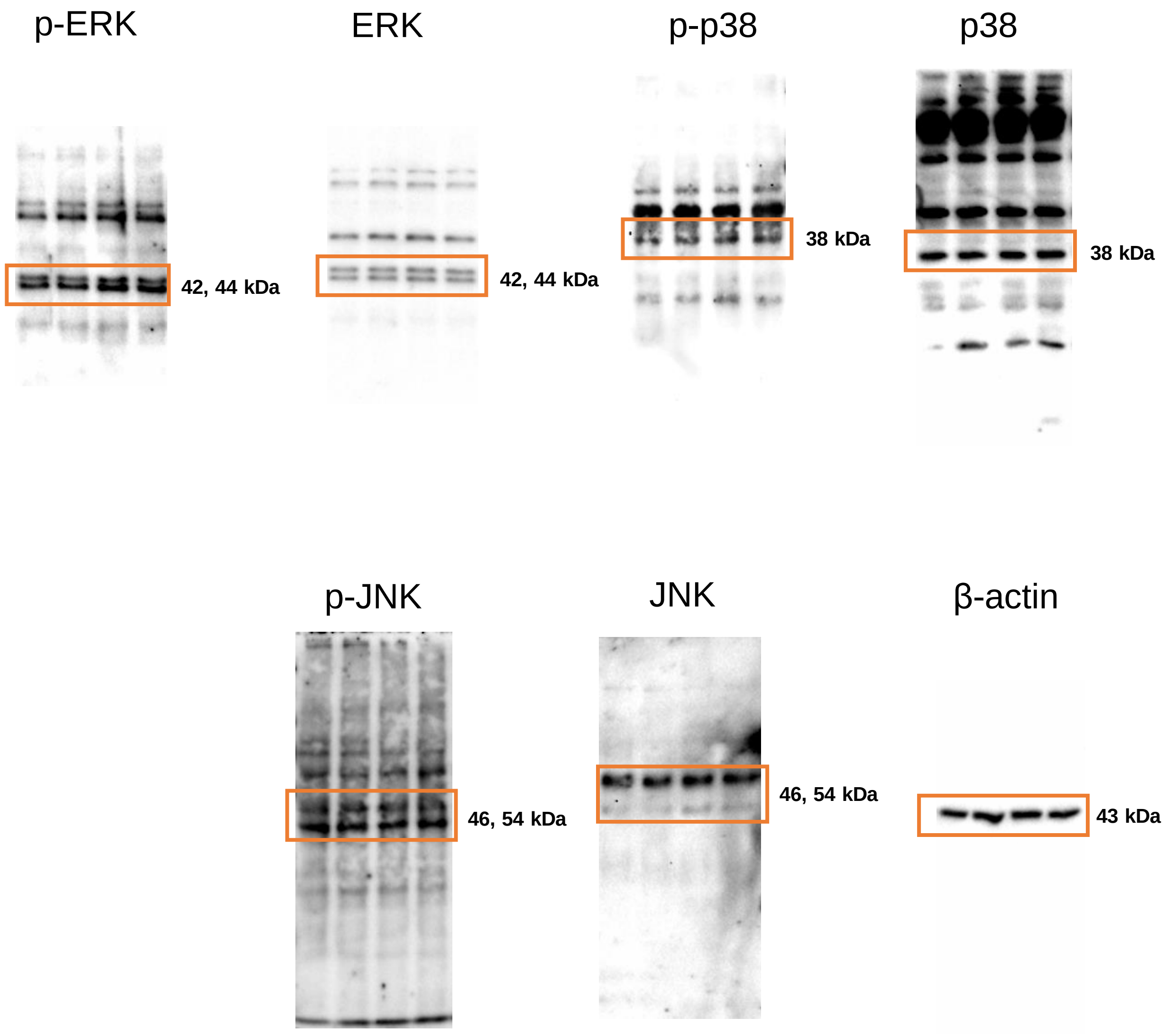

Supplemental Fig S3.

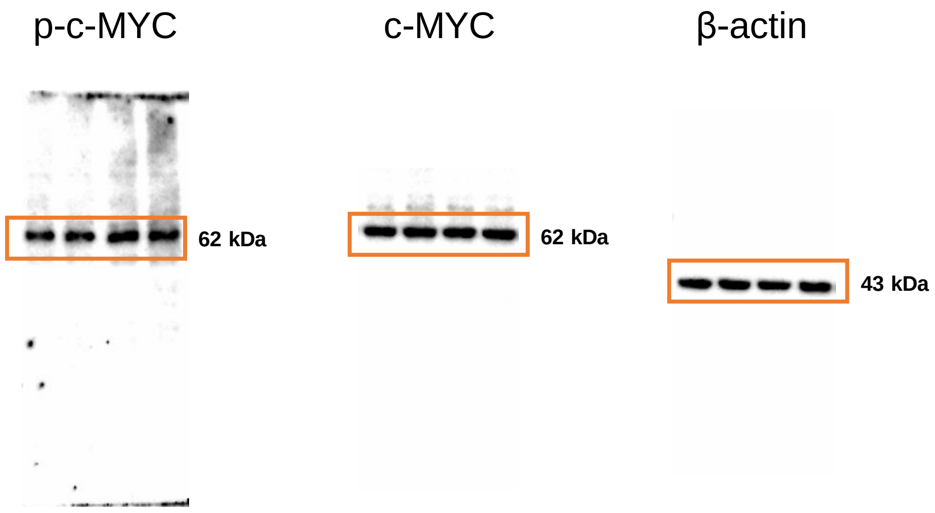

Supplement: Supplementary file 3 — Supplemental Material uncropped WB gel [file 41419_2023_5676_MOESM3_ESM.pdf]
